# Integrated Computational and Functional Screening Identifies G9a Inhibitors for *SETD2*-mutant Leukemia

**DOI:** 10.1093/gpbjnl/qzaf035

**Published:** 2025-04-29

**Authors:** Ya Zhang, Mengfang Xia, Zhenyi Yi, Pinpin Sui, Xudong He, Liping Wang, Qiyi Chen, Hong-Hu Zhu, Gang Huang, Qian-Fei Wang

**Affiliations:** China National Center for Bioinformation, Beijing 100101, China; Beijing Institute of Genomics, Chinese Academy of Sciences, Beijing 100101, China; University of Chinese Academy of Sciences, Beijing 100049, China; China National Center for Bioinformation, Beijing 100101, China; Beijing Institute of Genomics, Chinese Academy of Sciences, Beijing 100101, China; University of Chinese Academy of Sciences, Beijing 100049, China; China National Center for Bioinformation, Beijing 100101, China; Beijing Institute of Genomics, Chinese Academy of Sciences, Beijing 100101, China; University of Chinese Academy of Sciences, Beijing 100049, China; China National Center for Bioinformation, Beijing 100101, China; Beijing Institute of Genomics, Chinese Academy of Sciences, Beijing 100101, China; China National Center for Bioinformation, Beijing 100101, China; Beijing Institute of Genomics, Chinese Academy of Sciences, Beijing 100101, China; University of Chinese Academy of Sciences, Beijing 100049, China; China National Center for Bioinformation, Beijing 100101, China; Beijing Institute of Genomics, Chinese Academy of Sciences, Beijing 100101, China; University of Chinese Academy of Sciences, Beijing 100049, China; China National Center for Bioinformation, Beijing 100101, China; Beijing Institute of Genomics, Chinese Academy of Sciences, Beijing 100101, China; University of Chinese Academy of Sciences, Beijing 100049, China; Department of Hematology, Beijing Chao-Yang Hospital, Capital Medical University, Beijing 100020, China; Chinese Institutes for Medical Research, Beijing 100069, China; Department of Cell Systems and Anatomy, University of Texas Health San Antonio, San Antonio, TX 78229, USA; China National Center for Bioinformation, Beijing 100101, China; Beijing Institute of Genomics, Chinese Academy of Sciences, Beijing 100101, China; University of Chinese Academy of Sciences, Beijing 100049, China

**Keywords:** *SETD2-*mutant leukemia, G9a inhibitor, L1000, MYC, let-7a

## Abstract

*SETD2*, a frequently mutated epigenetic tumor suppressor gene in acute leukemia, is associated with chemotherapy resistance and poor patient outcomes. To explore potential therapeutics for *SETD2-*mutant leukemia, we employed an integrated approach combining computational prediction with epigenetic compound library screening. This approach identified G9a inhibitors as promising candidates, capable of reversing gene expression signatures associated with *Setd2* deficiency and selectively inhibiting *SETD2*-deficient cells. RNA sequencing analysis revealed that the G9a inhibitor significantly downregulated *Myc* and *Myc*-regulated genes involved in translation, DNA replication, and G1/S transition in *Setd2*-mutant cells. Further chromatin immunoprecipitation sequencing analysis showed that G9a inhibition reduced H3K9me2 levels at the long non-coding RNA *Mir100hg* locus, coinciding with specific upregulation of the embedded microRNA let-7a-2 in *Setd2*-mutant cells. Given the established role of let-7a in MYC suppression, these findings suggest a potential mechanism by which G9a inhibitors induce MYC downregulation in *SETD2-*mutant leukemia. Additionally, correlation analysis between computational predictions and phenotypic outcomes highlighted the MYC signature as a key predictor of drug efficacy. Collectively, our study identifies G9a inhibitors as a promising therapeutic avenue for *SETD2*-mutant leukemia and provides novel insights into refining drug prediction strategies.

## Graphical abstract

**Graphical abstract:**
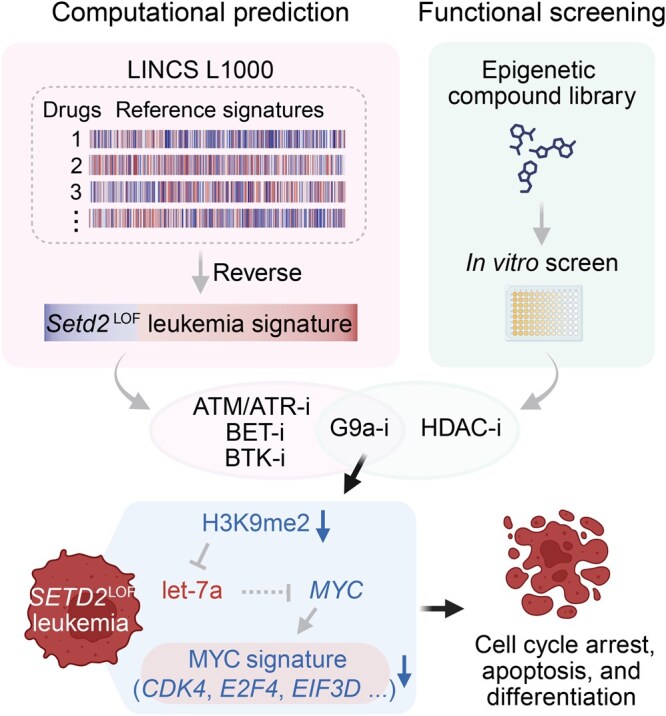

## Introduction

The histone H3K36me3 methyltransferase *SETD2* is a critical tumor suppressor gene that has been found to harbor loss-of-function (LOF) mutations or exhibit reduced expression in a wide range of human tumors [1–4]. Previously, we identified *SETD2* LOF mutations in approximately 6% of acute myeloid leukemia (AML) and acute lymphoid leukemia (ALL), with a particularly high prevalence of over 20% in mixed lineage leukemia-rearranged (MLL-r) leukemia [5]. These mutations often confer chemotherapy resistance, significantly impacting the prognosis and long-term survival of patients [6–8]. To address this issue, several inhibitors have been proposed to restore chemotherapy sensitivity in *SETD2-*deficient leukemic cells. These inhibitors include those targeting the H3K36 demethylase KDM4A and the G2/M cell cycle checkpoint regulators WEE1 and CHK1, which aim to address the impaired DNA damage response (DDR) resulting from SETD2 inactivation [6,7,9]. However, their therapeutic effects have been limited, as evidenced by the failure to prolong survival in mouse models. Consequently, there is an urgent need to explore alternative therapeutic strategies for *SETD2-*mutant leukemia.

As the sole H3K36me3 methyltransferase, SETD2 regulates various cellular processes beyond DDR, including transcriptional activation and elongation, alternative splicing, and interactions with other epigenetic modifications such as H3K79me2 and H3K27me3 [1,10–13]. In *SETD2* LOF mutations, these processes are often dysregulated, resulting in aberrant gene expression and contributing to the aggressiveness of *SETD2*-mutant leukemia. In this context, computational methods have emerged as a promising approach for identifying drugs that can reverse disease-associated molecular states [14–16]. By leveraging gene expression profiles as disease signatures and utilizing large-scale databases such as LINCS L1000 (a database containing over 1 million drug-response transcriptional profiles), researchers have demonstrated the potential of this strategy in drug discovery [17–19]. Based on these findings, we hypothesized that this approach could be applied to identify potential drugs for *SETD2*-mutant leukemia. However, while the inclusion of well-studied or FDA-approved drugs in these databases offers a cost-effective and accelerated path to drug discovery, the accuracy of predictions remains limited by factors such as disease signature representation and cell line heterogeneity. Therefore, further validation is necessary to confirm the efficacy of candidate drugs [20,21]. Moreover, considering the limited variety of epigenetic drugs in the database and potential crosstalk among H3K36me3 and other epigenetic modifications, additional exploration of these drugs in *SETD2-*mutant leukemia is warranted.

In this study, we integrated computational prediction with epigenetic compound library screening to systematically identify potential therapeutic agents for *SETD2*-mutant leukemia. By leveraging the LINCS L1000 database, we identified four pharmacologic classes capable of reversing *SETD2*-deficient transcriptional signatures: ATM/ATR inhibitors (ATM/ATR-i), bromodomain and extraterminal inhibitors (BET-i), Bruton’s tyrosine kinase inhibitors (BTK-i), and G9a inhibitors (G9a-i). Notably, G9a-i were also identified in the epigenetic compound library screening, showing increased sensitivity in *SETD2*-deficient leukemic cells compared to wild-type cells, prompting their selection for further investigation. Mechanism studies revealed that G9a-i significantly downregulated MYC and its target genes in *SETD2*-deficient cells. This effect is likely mediated by reduced H3K9me2 levels at the long non-coding RNA (lncRNA) *MIR100HG* locus, which leads to the specific upregulation of the embedded microRNA (miRNA) let-7a-2, a known repressor of MYC [22,23], in *SETD2*-deficient cells. Finally, comparative analysis of gene expression reversal patterns among the predicted candidates highlighted the MYC signature as a critical indicator of the phenotypic efficacy of the predicted drugs.

## Results

### Computational prediction of potential therapeutic candidates for *Setd2*-deficient leukemia

**Figure 1:**
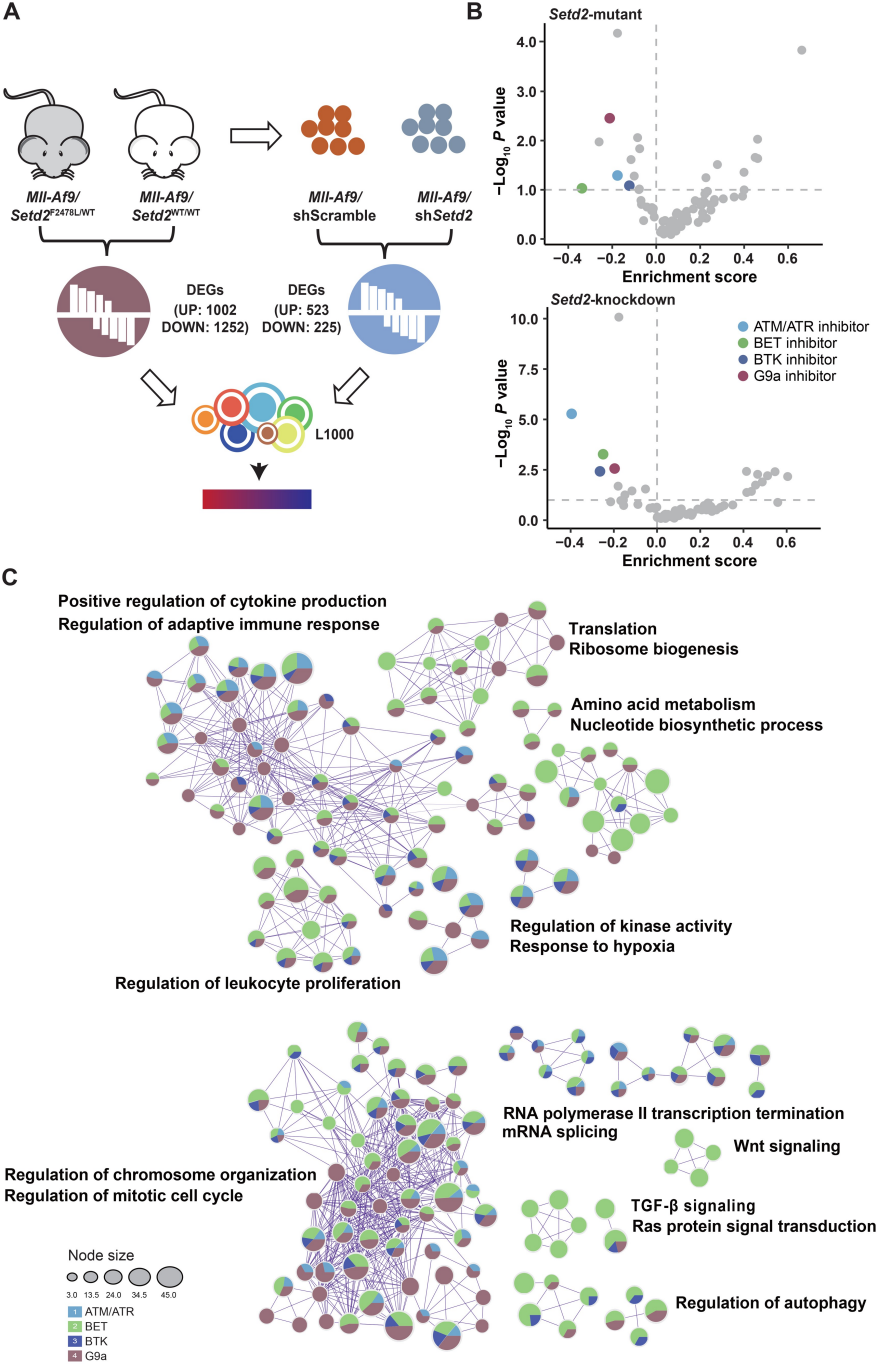
Top drug candidates identified by computational prediction for *Setd2*-deficient leukemia **A**. Schematic diagram of the computation prediction workflow based on *Setd2-*deficient leukemia signatures and drug expression profiles from the LINCS L1000 database. **B**. Dot plots illustrating the enrichment score and statistical significance of each drug class against *Setd2*-mutant (top) and *Setd2-*knockdown (bottom) leukemia signatures. ATM/ATR, BET, BTK, and G9a inhibitors are highlighted as overlapping candidates between both gene sets, exhibiting significant enrichment (enrichment score < 0, *P* ≤ 0.05). **C**. Enrichment network visualization of the biological functions of genes reversed by candidate drugs highlighted in (B) against *Setd2*-mutant leukemia signatures. Nodes are depicted as pie charts, indicating their associations with each drug class. Cluster labels were manually added, and colors denote the identities of each drug class. BET, bromodomain and extraterminal; BTK, Bruton’s tyrosine kinase.

Since transcriptomic data from leukemia patients with *SETD2* mutations are unavailable in public databases, we established the signature of *Setd2*-deficient leukemia using gene expression data from leukemic cells derived from previously generated mouse models. To improve the reproducibility of the prediction, two sets of differentially expressed genes (DEGs) from different conditions of *Setd2* LOF were collected: (1) *Mll-Af9/Setd2*^F2478L/WT^  *versus Mll-Af9/Setd2*^WT/WT^, where the *Setd2*-mutant allele corresponds to a mutation found in an AML patient [6]; (2) *Mll-Af9* with *Setd2* knockdown *versus* scramble control, as previously analyzed [12] (**Figure 1**A; Table S1). These two gene sets share similar biological pathways, with upregulated genes enriched in functions such as inflammatory response, cell migration, and cell activation, while downregulated genes are associated with cell division, cell cycle, and microtubule organization (Figure S1A and B).

Subsequently, we compared the *Setd2*-deficient leukemia signature with drug-response transcriptional profiles from the L1000 database to predict potential therapeutic agents, using a method adapted from previous studies [16,18]. Briefly, each transcriptional profile was assigned a reversal gene expression score (RGES) to quantify its reversal potency against the signature. A lower RGES indicates stronger reversal, while a higher RGES suggests greater similarity. To account for treatment variations, a summarized RGES (sRGES) was calculated for each drug, representing its overall reversal potency (Table S2). We observed that chemotherapies, including daunorubicin and doxorubicin, exhibited signatures similar to the dysregulated genes, supporting the chemo-resistant properties of *Setd2* LOF (Figures S1C, S1D, and S3A).

Currently, candidate drugs are typically selected based on their sRGES ranking [18]. However, there are two challenges: firstly, minimal variation in sRGES among top-ranked drugs complicates the selection process; secondly, the drug’s target may not be representative, hindering successful validation. To address these, we adopted a more robust strategy by focusing on drug classes rather than individual drugs [24]. Specifically, drugs sharing the same mechanism of action (MOA) were grouped into drug classes. We then performed drug enrichment analysis to prioritize drug classes in which member drugs significantly reversed the disease signatures (enrichment score < 0, *P* ≤ 0.05) (Table S2). Four drug classes overlapped between both sets of *Setd2*-deficient leukemia signatures, including ATM/ATR-i, BET-i, BTK-i, and G9a-i (Figure 1B). The majority of drugs in each drug class had negative sRGES values, supporting their reversal potency against the disease signatures (Figure S2A and B). However, we observed that different drug classes exhibited varying reversal tendencies (Figure 1C, Figure S3A and B). BET-i showed the broadest effect on disease signatures, reversing genes involved in multiple pathways. While all four drug classes reversed genes related to immune response, cell proliferation, chromosome organization, and mitotic cell cycle, BET-i and G9a-i also inhibited translation, nucleotide biosynthesis, and autophagy.

Previous study has shown that *SETD2-*mutant leukemia exhibits reduced phosphorylated ATR, indicating impaired DDR [6]; however, the efficacy of ATM/ATR-i in *SETD2*-mutant leukemia remains unknown. BTK, a key component of the B-cell receptor signaling pathway, has been targeted in B-cell malignancies, but its role in leukemia is unexplored [25]. BET proteins and G9a, both epigenetic regulators, play critical roles in transcription [26–28]. BET proteins recognize acetylated lysine residues on histones, modulating gene expression, while G9a methylates H3K9me2, typically associated with gene silencing. Given the gene reversal preferences, all these inhibitors were selected as potential therapeutics for further validation in *SETD2*-mutant leukemia.

### Epigenetic compound library screening identified G9a-i sensitizing *SETD2*-deficient leukemic cells

**Figure 2:**
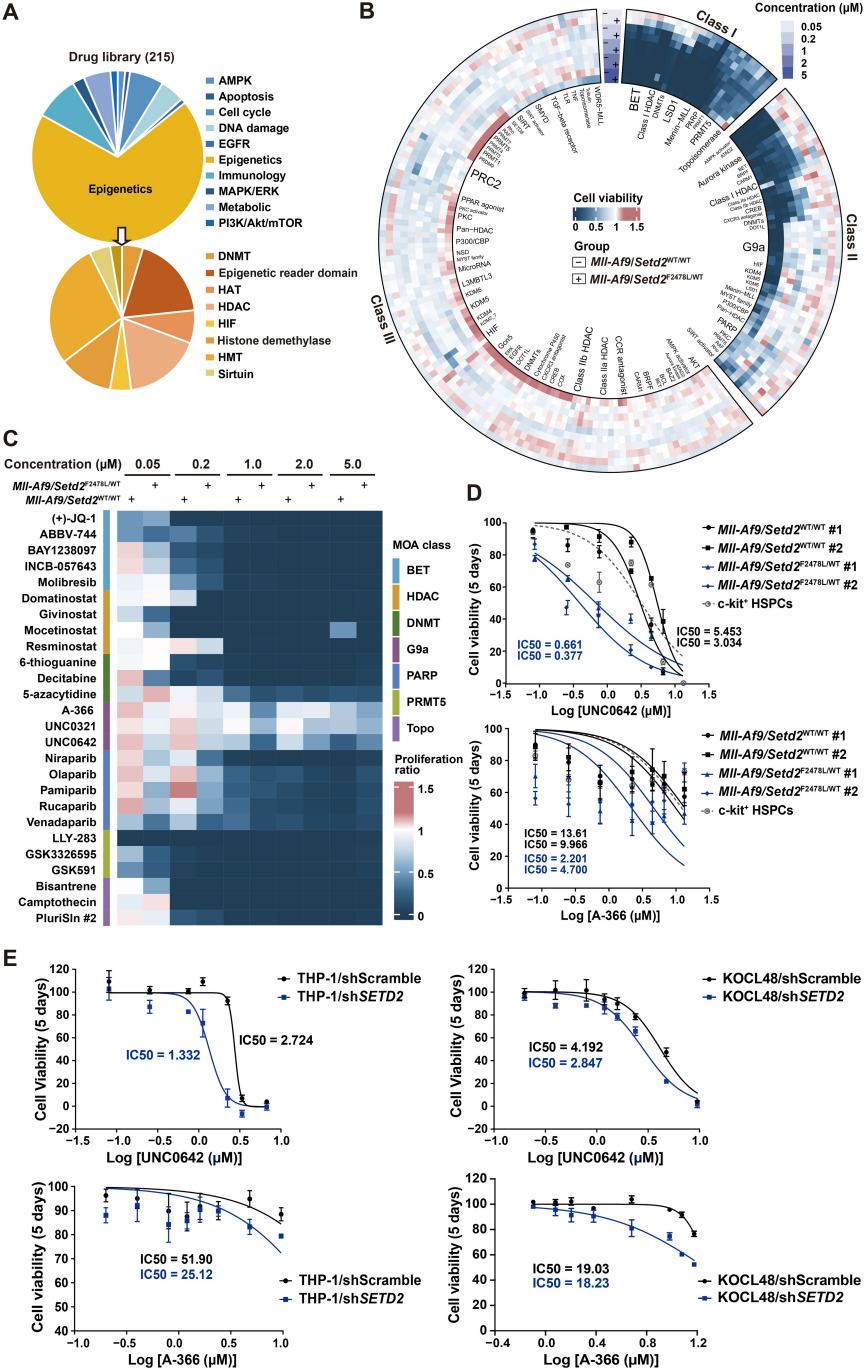
Epigenetic compound library screening and validation of drugs specifically inhibiting *Setd2*-mutant leukemic cells **A**. Classification summary of drugs in the epigenetic compound library. The library includes compounds targeting epigenetic enzymes and related factors. **B**. Heatmap showing the sensitivity of *Mll-Af9*/*Setd2*^WT/WT^ and *Mll-Af9*/*Setd2*^F2478L/WT^ cells to different concentrations (0.05, 0.2, 1, 2, and 5 µM) of compounds after 5 days of treatment. Three major classes were identified through clustering analysis, with drug targets labeled by font size proportional to their distribution frequency. The color bar indicates the normalized effect of compounds on cell viability inhibition (blue for inhibition and red for stimulation) relative to DMSO-treated controls. **C**. Heatmap showing the sensitivity of *Mll-Af9/Setd2*^WT/WT^ and *Mll-Af9*/*Setd2*^F2478L/WT^ cells to selected drugs that exhibit a trend of enhanced inhibitory effects on *Setd2*-mutant cells, as well as those with unclear differential effects between the two cell lines in (B). **D**. and **E**. Graphs showing the dose-response curves and IC50 values of G9a inhibitors UNC0642 (top) and A-366 (bottom) in *Mll-Af9/Setd2*^WT/WT^ and *Mll-Af9*/*Setd2*^F2478L/WT^ leukemic cells (D) and human leukemia cell lines (THP-1 and KOCL48) with scramble control or *SETD2* knockdown (E), treated with a range of concentrations for 5 days. Two biological replicates were performed for each genotype, with IC50 value shown for each cell line, and normal c-kit^+^ HSPCs included as cytotoxicity controls in (E). Data are shown as mean ± SEM (*n* = 3) in (D and E). HSPC, hematopoietic stem and progenitor cell; MOA, mechanism of action; SEM, standard error of the mean; WT, wild-type.

H3K36me3, mediated by SETD2, engages in extensive crosstalk with other epigenetic modifications, such as antagonism with H3K36me2 and H3K27me3, and recruitment of DNMT3B and histone acetyltransferases [1,10,29,30]. However, it remains unclear whether these modifications are elevated in *SETD2-*mutant leukemia and whether they represent potential therapeutic targets. Additionally, the epigenetic inhibitors available in the L1000 database are limited. Therefore, we further screened *Mll-Af9/Setd2*^F2478L/WT^ leukemic cells, along with *Mll-Af9* leukemic cells as control, to assess their sensitivity toward an epigenetic compound library. This library contained 215 inhibitors targeting epigenetic writers, erasers, readers, and related factors (**Figure 2**A; Table S3). Considering the wide range of IC50 values [from nanomolar (nM) to micromolar (µM)], we tested five concentrations (0.05, 0.2, 1, 2, and 5 µM). Unsupervised hierarchical clustering revealed three primary response patterns to the treatment (Figure 2B, Figure S4). The first group comprised inhibitors that suppressed growth in both *Setd2-*mutant and wild-type leukemic cells at least at four or more concentrations. These mainly included BET-i, LSD1 inhibitors (LSD1-i), Menin-MLL inhibitors (Menin-MLL-i), PRMT5 inhibitors (PRMT5-i), and topoisomerase inhibitors (Topo-i). Notably, LSD1-i and Menin-MLL-i consistently inhibited cell growth by 70%–80% across all concentrations, regardless of genotype. This supports their established efficacy against MLL-r leukemia [31–34] and indicates that *SETD2* mutation does not affect their potency. The second group consisted of compounds that showed dose-dependent inhibition in *Mll-Af9* and/or *Mll-Af9/Setd2*^F2478L/WT^ cells. Examples include G9a-i, class I HDAC inhibitors (class I HDAC-i), and PARP inhibitors (PARP-i). The third group included inhibitors with minimal or no effect on these cells, such as PRC2 inhibitors and class II HDAC inhibitors, suggesting they are unsuitable for treating MLL-r leukemia, irrespective of *SETD2* mutation status.

Our aim was to identify inhibitors that exhibit hypersensitivity in *SETD2-*mutant leukemia. Based on clustering results, we selected inhibitors from cluster I and cluster II that showed a trend of stronger inhibitory effects on *Setd2*-mutant cells, as well as those with inconclusive differences between the two cell lines, for further validation (Figure 2C; Table S3). We conducted dose-response experiments with these inhibitors, including G9a-i (*e.g.*, UNC0642 and A366), BET-i (*e.g.*, molibresib and INCB-057643), and PRMT5-i (*e.g.*, GSK591 and GSK3326595), among others. The results revealed that G9a-i exhibited significantly stronger inhibitory effects on *Setd2-*mutant cells (Figure 2D). In contrast, most other inhibitors, such as BET-i and PARP-i, showed no notable sensitivity differences between the two genotypes, except for mocetinostat, one of the class I HDAC-i (Figure S5A–D). Although BET-i were predicted as potential therapeutics for *Setd2-*mutant cells, our results suggest that their gene reversal effects might be excessive, as evidenced by the severe inhibition of normal c-kit^+^ hematopoietic stem and progenitor cells (HSPCs). Notably, *Setd2*-mutant leukemic cells showed greater resistance to PRMT5-i and Topo-i compared to *Setd2* wild-type cells (Figure S5E and F). Given the promising pre-clinical results of PRMT5-i in leukemia [35–37], our findings suggest that *SETD2* mutation status should be considered in treatment strategies. We also tested ATM/ATR-i and BTK-i, which were computationally predicted but not included in the library; however, no significant sensitivity differences were observed between *Setd2*-mutant and wild-type cells (Figure S6A and B). The sensitivity to G9a-i was recapitulated in human THP-1 and KOCL48 cell lines with *SETD2* knockdown relative to scramble controls (Figure 2E, Figure S6C), while THP-1/sh*SETD2* and KOCL48/sh*SETD2* also showed chemo-resistance as expected (Figure S6D).

Overall, the epigenetic compound library screening identified G9a-i as potential drugs for *SETD2-*deficient cells, which were also prioritized by the computational prediction.

### UNC0642 induced apoptosis and cell cycle arrest and inhibited self-renewal in *Setd2*-mutant leukemic cells

**Figure 3:**
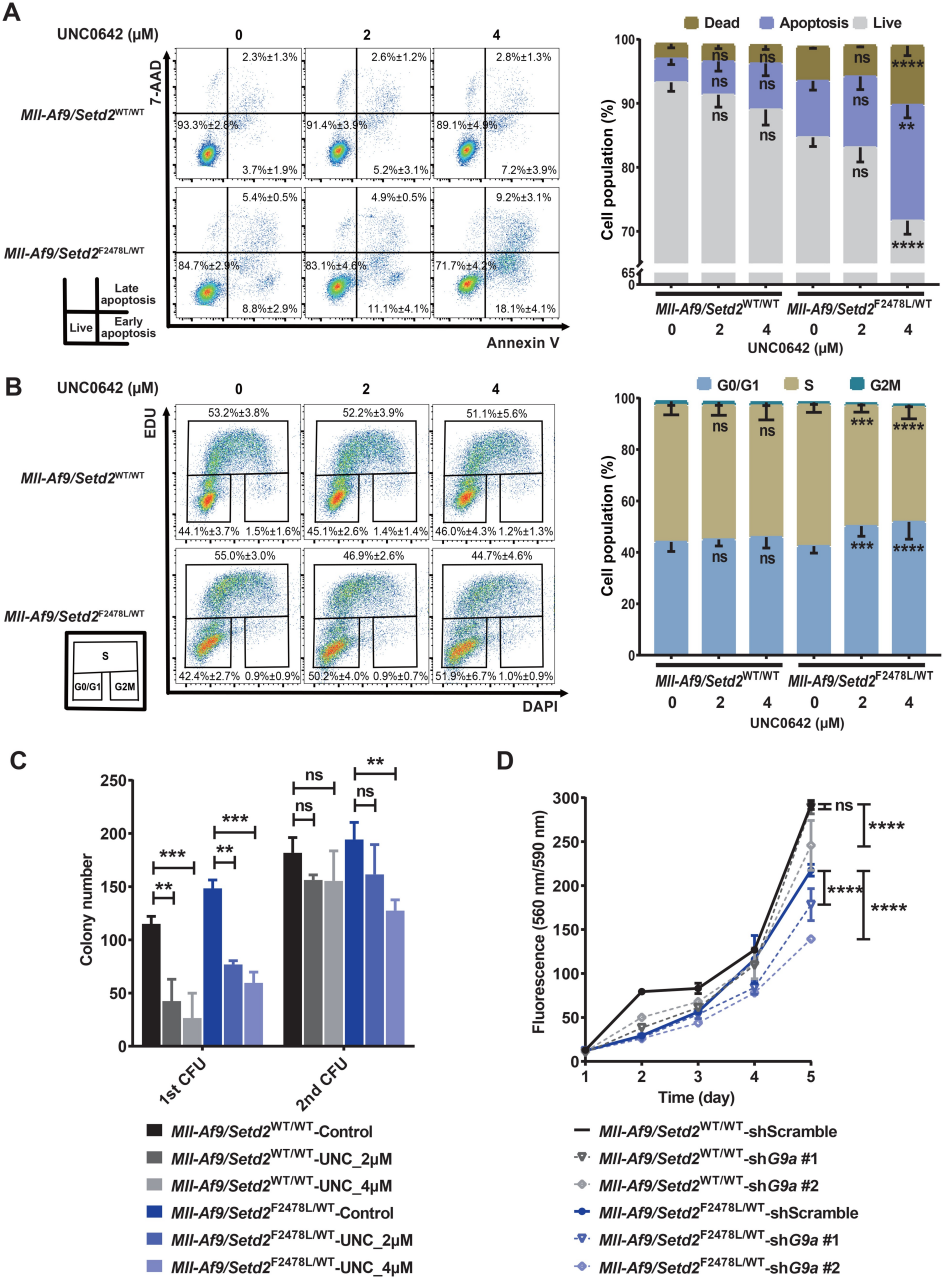
UNC0642 induced apoptosis and cell cycle arrest and inhibited self-renewal of *Setd2*-mutant leukemic cells *Mll-Af9/Setd2* ^WT/WT^ and *Mll-Af9*/*Setd2*^F2478L/WT^ cells were treated with UNC0642 (2 µM and 4 µM) or DMSO control (represented as 0 µM) for 72 h in (A–C). **A**. Left: representative dot plots of cell apoptosis. The population definition of live, early apoptotic, and late apoptotic cells is shown in the bottom left. Right: graphical representation of the corresponding statistical results. **B**. Left: representative dot plots of cell cycle phase distribution. The population definition of G0/G1, S, and G2/M phases is shown in the bottom left. Right: graphical representation of the corresponding statistical results. **C**. Bar plot showing colony number from the colony-forming assay in serial re-plating. **D**. Line chart showing the proliferation state of *Mll-Af9/Setd2*^WT/WT^ and *Mll-Af9*/*Setd2*^F2478L/WT^ cells with *G9a* knockdown and scramble control. Data are shown as mean ± SEM (*n* = 3). **, *P* < 0.01; ***, *P* < 0.001; ****, *P* < 0.0001; ns, not significant (two-way ANOVA test). CFU, colony-forming unit.

Next, we investigated the role of G9a-i in inhibiting the proliferation of *Setd2*-mutant leukemic cells. UNC0642 was selected for further exploration due to its higher selectivity and lower toxicity [38]. Its on-target effect was confirmed by a dose-dependent reduction in H3K9me2 level (Figure S7A). Subsequent flow cytometric analysis revealed that UNC0642 induced more significant apoptosis and G1-to-S phase cell cycle arrest in *Mll-Af9/Setd2*^F2478L/WT^ cells compared with *Mll-Af9* cells (**Figure 3**A and B). The serial colony-forming unit (CFU) assay showed that pre-treatment with UNC0642 for 3 days persistently inhibited the colony formation of *Setd2*-mutant cells for two passages, while it only inhibited *Mll-Af9* cells in the first passage (Figure 3C). This suggests that the self-renewal capacity of *Setd2*-mutant cells was more profoundly suppressed. Supportingly, UNC0642 promoted the differentiation of *Setd2*-mutant cells more significantly than *Setd2* wild-type cells, as indicated by increased levels of the mature granulocyte and monocyte marker CD11b (Figure S7B). To further validate the effects of G9a inhibition, we knocked down *G9a* in *Mll-Af9/Setd2*^F2478L/WT^ and *Mll-Af9* cells using two independent lentiviral shRNAs (Figure S7C and D). As expected, *G9a* knockdown inhibited the growth of *Setd2*-mutant cells more significantly compared to wild-type cells (Figure 3D).

### UNC0642 suppressed the MYC signature in *SETD2*-deficient cells

We further explored the mechanisms underlying the hypersensitivity of *Setd2*-mutant cells to G9a-i. Previous studies have suggested that G9a is overexpressed in certain cancers, rendering them sensitive to G9a-i. We hypothesized that this hypersensitivity might be due to either higher levels of G9a in *Setd2*-mutant cells or an unknown crosstalk between H3K9me2 and H3K36me3. However, both the RNA and protein levels of G9a, as well as the histone mark H3K9me2, showed no difference between *Setd2*-mutant and wild-type cells (Figure S8A and B). Additionally, treatment with G9a-i did not affect H3K36me3 levels (Figure S7A). These findings suggest that the sensitivity difference was not attributable to these factors.

**Figure 4:**
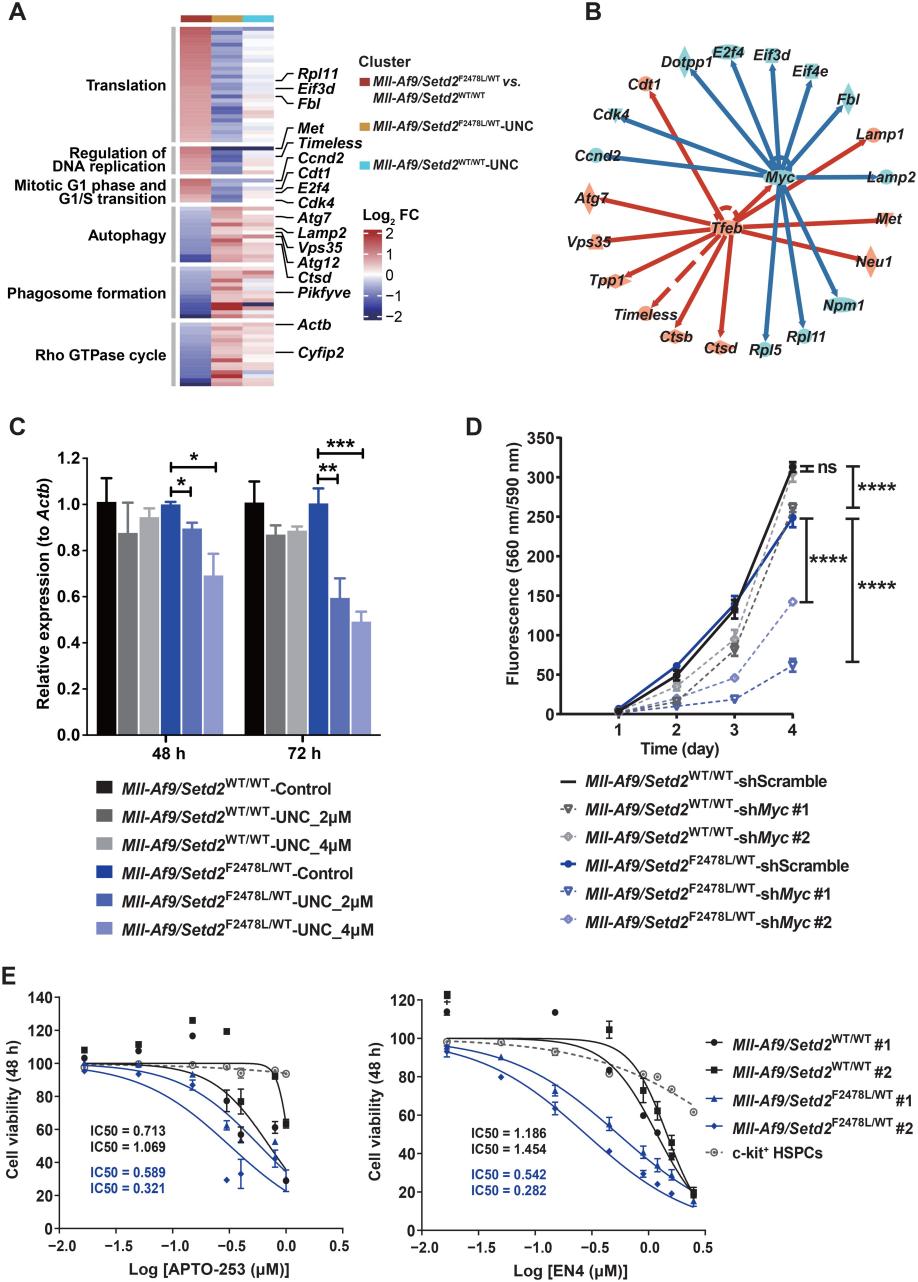
UNC0642 repressed Myc in *Setd2*-mutant cells **A**. Heatmap displaying genes in the top active and repressed canonical pathways from IPA of reversed DEGs in *Mll-Af9*/*Setd2*^F2478L/WT^ cells under UNC0642 treatment against its signature. Genes in each term were ranked and color-coded (red for up, blue for down) by FC in *Setd2*-mutant leukemia signature and then re-color-coded by FC after UNC0642 treatment in *Mll-Af9*/*Setd2*^F2478L/WT^ and *Mll-Af9/Setd2*^WT/WT^ cells respectively. **B**. Regulatory network showing top upstream regulators and their targets predicted to be activated or suppressed in reversed DEGs. Colors indicate increased (red) or decreased (blue) gene expression under UNC0642 treatment. Red and blue lines represent known activating or inhibitory effects, respectively, between each regulator and its targets. **C**. Bar plot showing the mRNA levels of *Myc* in *Mll-Af9/Setd2*^WT/WT^ and* Mll-Af9*/*Setd2*^F2478L/WT^ cells treated with 2 µM and 4 µM UNC0642 relative to DMSO control at 48 h and 72 h. **D**. Line chart showing the proliferation state of *Mll-Af9/Setd2*^WT/WT^ and *Mll-Af9*/*Setd2*^F2478L/WT^ cells with *G9a* knockdown and scramble control. **E**. Graphs showing the dose-response curves and IC50 values of MYC inhibitors APTO-253 and EN4 in *Mll-Af9/Setd2*^WT/WT^ and* Mll-Af9*/*Setd2*^F2478L/WT^ leukemic cells treated with variable concentrations for 48 h. Two biological replicates were performed for each genotype, and IC50 values are shown for each cell line, with normal c-kit^+^ HSPCs included as cytotoxicity controls. In (C–E), data are shown as mean ± SEM (*n* = 3). *, *P* < 0.05; **, *P* < 0.01; ***, *P* < 0.001; ****, *P* < 0.0001; ns, not significant (two-way ANOVA test). IPA, Ingenuity Pathway Analysis; DEG, differentially expressed gene; FC, fold change.

To elucidate the potential molecular mechanisms, we performed RNA sequencing (RNA-seq) analysis on UNC0642-treated *Mll-Af9/Setd2*^F2478L/WT^ and *Mll-Af9* cells. Differential expression analysis revealed that UNC0642 induced more DEGs in *Setd2*-mutant cells compared to wild-type cells (Table S4). Upregulated genes were involved in lysosome, endocytosis, inflammatory, *etc.*, while downregulated genes were enriched in ribonucleoprotein complex biogenesis, cell cycle, and DNA metabolic process (Figure S8C). We further focused on genes reversed by UNC0642 in *Setd2-*mutant cells, where UNC0642 repressed 114 upregulated genes and induced 244 downregulated genes. Ingenuity Pathway Analysis (IPA) showed that these genes were related to the translation repression (*e.g.*, *Eif3d* and *Eif4h*), DNA replication (*e.g.*, *Met* and *Timeless*), and G1/S transition (*e.g.*, *Cdk4* and *E2f4*), as well as activation of autophagy (*e.g.*, *Atg7* and *Vps35*), phagosome formation (*e.g.*, *Pikfyve*), and the Rho GTPase cycle (*e.g.*, *Actb* and *Cyfip2*), with more significant changes in *Setd2-*mutant cells relative to wild-type cells (**Figure 4**A; Table S5). Upstream regulator analysis identified *Myc* as the most prominently suppressed hub, associated with translation suppression and G1/S transition, and *Tfeb* as the major activated hub, linked to the upregulation of autophagy (Figure 4B; Table S5). Given that phagosome formation and Rho GTPase are closely related to autophagy, they were expected to co-regulate with autophagy after UNC0642 treatment. Consistently, gene set enrichment analysis (GSEA) showed that UNC0642 more significantly repressed MYC-regulated genes and activated lysosome-related genes, which play key roles in autophagy, in *Setd2*-mutant cells compared with wild-type cells (Figure S8D). Furthermore, the downregulation of Myc expression, confirmed by reverse transcription quantitative PCR (RT-qPCR) and Western blot, and the induction of autophagy, also confirmed by Western blot, were both more pronounced in *Setd2*-mutant cells (Figure 4C, Figure S8E).

We further investigated the contributions of these two roles of G9a-i in mediating its effectiveness in *Setd2*-mutant cells. *Myc* knockdown more significantly inhibited the proliferation of *Setd2*-mutant cells compared to *Mll-Af9* cells (Figure 4D). Moreover, MYC inhibitors APTO-253 and EN4 recapitulated the differential sensitivity observed with UNC0642 between *Setd2*-mutant and wild-type cells (Figure 4E). In contrast, autophagy inhibitors 3-methyladenine (3-MA) and autophinib only slightly reversed the proliferation inhibition induced by UNC0642 in *Setd2-*mutant cells (Figure S8F). These results suggest that Myc downregulation, mediated by G9a inhibition, plays a more critical role in the survival of *Setd2*-mutant cells. The downregulation of the MYC signature by UNC0642 was further validated in *SETD2*-deficient human cell lines. Specifically, *MYC* and its downstream target genes (*e.g.*, *CDK4*, *E2F4*, *EIF3D*, and *RPL11*) were more significantly suppressed in THP-1/sh*SETD2* and KOCL48/sh*SETD2* cells compared to scramble controls (Figure S9). Together, these findings demonstrate that the G9a inhibitor UNC0642 more potently represses the MYC signature in *SETD2*-deficient cells, which is critical for their survival.

### UNC0642 induced let-7a-2 in *SETD2*-deficient cells

**Figure 5:**
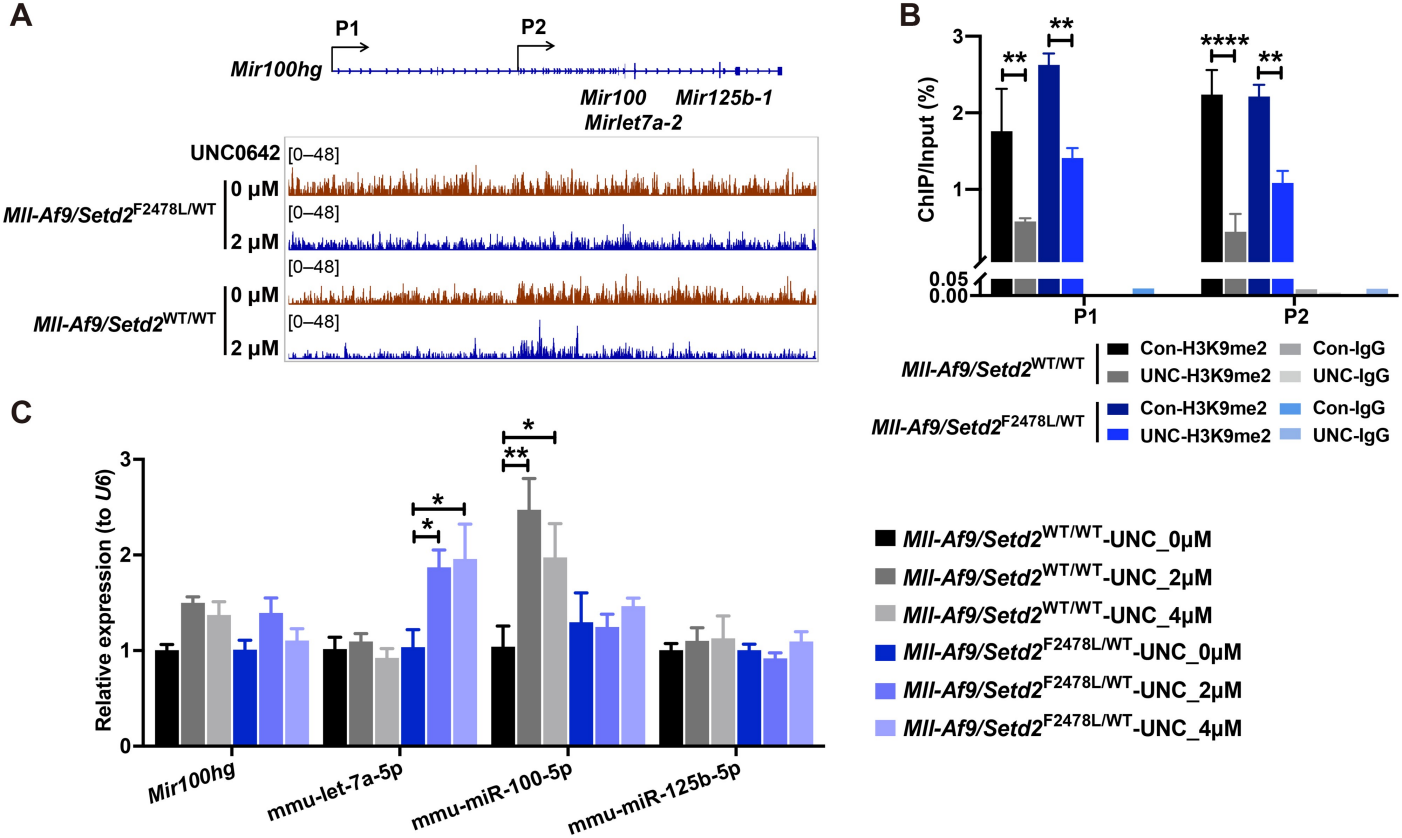
UNC0642 induced let-7a-2 in *Setd2*-mutant cells **A**. Schematic representation of the *Mir100hg* genomic locus (top) and IGV visualization of H3K9me2 levels at the *Mir100hg* genomic locus (bottom). **B**. Bar plot showing changes in H3K9me2 levels at two potential promoter sites of the *Mir100hg* locus, normalized to input. In (A and B), *Mll-Af9/Setd2*^WT/WT^ and *Mll-Af9/Setd2*^F2478L/WT^ cells were treated with 2 µM UNC0642 or DMSO control (represented as 0 µM) for 72 h. **C**. Bar plot showing expression changes of *Mir100hg* and the mature forms of its embedded miRNAs (mmu-let-7a-5p, mmu-miR-100-5p, and mmu-miR-125b-5p) after treatment with 2 µM and 4 µM UNC0642 for 48 h, relative to DMSO control (represented as 0 µM). Data are shown as mean ± SEM (*n* = 3). *, *P* < 0.05; **, *P* < 0.01; ****, *P* < 0.0001 (two-way ANOVA test). IGV, Integrative Genomics Viewer; ChIP, chromatin immunoprecipitation.

Given that G9a primarily mediates H3K9me2 modification, a marker of transcriptional repression, we hypothesized that G9a inhibition might suppress MYC through an indirect mechanism. To investigate this, we performed H3K9me2 chromatin immunoprecipitation sequencing (ChIP-seq) in *Mll-Af9/Setd2*^F2478L/WT^ and *Mll-Af9* cells treated with UNC0642. We then analyzed genes with reduced H3K9me2 levels for overlap with significantly upregulated genes identified by RNA-seq in both cell types (Tables S4 and S6). However, no common or known direct upstream regulators of Myc were identified. Since the RNA-seq data were obtained at a single timepoint (72 h), which might not fully capture dynamic gene expression changes, we speculated that the upstream regulators predicted by IPA could reflect earlier events. By further integrating genes with reduced H3K9me2 levels and IPA-predicted upstream regulators from all DEGs in the RNA-seq data (Table S7), we focused on the lncRNA *Mir100hg*, which is also known as the miR-100-let-7a-2-miR-125b-1 cluster host gene. We observed decreased H3K9me2 levels at the *Mir100hg* locus in both *Setd2*-mutant and wild-type cells (**Figure 5**A). Notably, let-7a-5p, the mature form of let-7a-2 [39], was predicted to be specifically activated in *Setd2*-mutant cells (Table S7). Previous studies have shown that let-7a (including let-7a-1, -2, and -3, which share identical sequences) binds to the 3ʹ UTR of *MYC*, leading to *MYC* mRNA degradation and translational repression [22,23]. Since let-7a-2 is embedded within *Mir100hg*, we propose that Myc downregulation in *Setd2*-mutant cells may result from the upregulation of let-7a-2, driven by decreased H3K9me2 levels at *Mir100hg.*

To validate these findings, we performed ChIP-qPCR, which confirmed the reduction in H3K9me2 modification at potential promoter regions of *Mir100hg* (Figure 5B). RT-qPCR demonstrated the upregulation of *Mir100hg*, as well as its embedded miRNAs miR-100 and let-7a-2, but not miR-125b-1 (detected by primers specific for their mature forms). Notably, we observed an upregulation of let-7a-2 in *Setd2*-mutant cells but not in wild-type cells, while the other two miRNAs did not show this specificity (Figure 5C). These results were further corroborated in human THP-1 and KOCL48 cells (Figure S10A and B). Consistent with previous studies showing that let-7a leads to *MYC* mRNA degradation and translational repression, our findings suggest that the specific upregulation of let-7a-2 is likely responsible for MYC suppression mediated by G9a-i in *SETD2*-deficient cells.

### Primary patient leukemic cells with *SETD2* mutations exhibited sensitivity to UNC0642 *in vitro*

While G9a-i demonstrated promising effects in *Setd2-*mutant mouse cells and *SETD2-*knockdown human cell lines, we further validated their efficacy in primary patient leukemic cells. Colony-forming assays revealed that the G9a inhibitor UNC0642 exerted stronger inhibitory effects in *SETD2-*mutant leukemic cells, as evidenced by reduced colony numbers and total cell counts (Figure S11A). Additionally, upregulation of let-7a and downregulation of the MYC signature were observed in *SETD2-*mutant leukemic cells (Figure S11B and C). These findings further underscore the therapeutic potential of G9a-i for treating *SETD2*-mutant leukemia.

### The MYC signature is essential to predict the actual efficacy of predicted drugs

**Figure 6:**
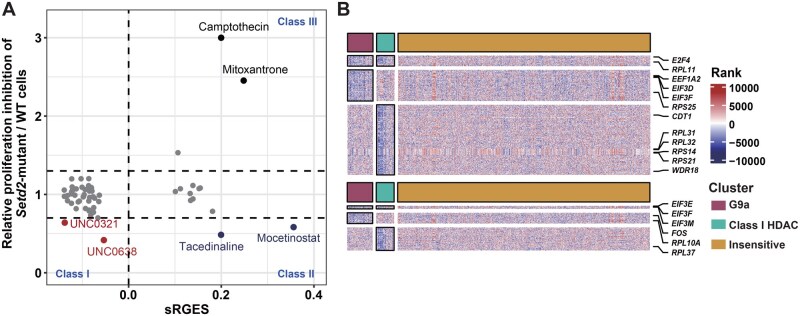
Correlation between computational prediction and phenotypic outcomes **A**. Scatter plot showing the correlation between phenotypic response (FC in cell proliferation inhibition between *Setd2*-mutant and wild-type cells) and predicted transcriptional response (sRGES) for overlapping drugs identified in the compound screening and computational prediction. Class I: responsive by both measures; Class II: responsive by sensitivity but not by L1000 sRGES; Class III: non-responsive by both measures. **B**. Heatmap displaying genes specifically reversed by compounds to which *Setd2*-mutant cells are sensitive (G9a inhibitors and class I HDAC inhibitors), compared with insensitive drugs (ATM/ATR inhibitors and BTK inhibitors). Upregulated and downregulated genes were ranked and color-coded by FC in the L1000 transcriptome profiles (red for up, blue for down), with *MYC* target genes reversed by UNC0642 highlighted. sRGES, summarized reversal gene expression score.

Considering that only G9a-i demonstrated specific sensitivity in *Setd2-*mutant cells among the predicted candidate drugs, we further investigated the correlation between computational predictions and phenotypic outcomes. To establish this correlation, we selected individual drugs that overlapped between the two strategies as representative examples. The molecular reversal level was quantified using sRGES, while the phenotypic response was measured by the fold change (FC) in cell proliferation inhibition between *Setd2*-mutant and wild-type cells. Most of these drugs showed only subtle differences in proliferation inhibition between the two cell lines, regardless of whether their sRGES values were positive or negative. The remaining drugs primarily exhibited three patterns: (1) drugs with negative sRGES values and increased sensitivity in *Setd2-*mutant cells, which included two G9a-i; (2) drugs with positive sRGES values but unexpectedly increased sensitivity in *Setd2-*mutant cells, both of which were class I HDAC-i; (3) drugs with positive sRGES values and resistance in *Setd2*-mutant cells, which were primarily Topo-i (**Figure 6**A). Interestingly, in the prediction, Topo-i were enriched as the top-ranked drugs among all candidates with signatures similar to *Setd2*-deficient cells, demonstrating that the drug enrichment method effectively facilitated the selection of drugs that better align with expectations (Table S2). Class I HDAC-i have been reported to upregulate inflammatory signaling and downregulate cell cycle-related genes [40,41], exhibiting a signature similar to *Setd2-*mutant cells. However, some studies also suggest that these inhibitors may suppress MYC pathways [42], which could explain their efficacy in *Setd2*-mutant cells (Figure S5C).

Since these drug candidates exhibited distinct gene reversal patterns (Figure 1C, Figure S3B), we further identified genes specifically reversed by sensitive drugs, such as G9a-i and class I HDAC-i, compared to insensitive drugs like ATM/ATR-i and BTK-i. BET-i were excluded from the analysis because they might excessively reverse disease-related genes. The results demonstrated that G9a-i and class I HDAC-i significantly suppressed MYC (Myc) target genes, including *E2F4* (*E2f4*), *EIF3D* (*Eif3d*),* EIF3F* (*Eif3f*),* CDT1* (*Cdt1*), and *RPS21* (*Rps21*) (Figure 6B). Together, these findings support the conclusion that the MYC signature, a subset of the *SETD2* LOF leukemia transcriptomic signatures, is a critical indicator of the actual efficacy of the predicted drug candidates.

## Discussion

*SETD2-*mutant leukemia is known for its aggressiveness and chemo-resistance, yet effective therapeutic options remain limited. By integrating computational prediction with epigenetic compound library screening, followed by *in vitro* validation, we identified G9a-i as a promising new therapeutic strategy. Mechanistically, G9a-i likely reduce H3K9me2 levels at the *MIR100HG* locus, specifically upregulating the embedded miRNA let-7a-2 to suppress the MYC pathway, which is crucial for the survival of *SETD2*-mutant leukemia. Additionally, analysis of drug-sensitive genes in *Setd2*-mutant cells highlighted the MYC signature as a key predictor of drug efficacy.

Compared to targeted therapies developed based on prior knowledge of cancer vulnerabilities, computational prediction provides a novel paradigm for drug discovery. Using the L1000 database, we identified drugs with varying potency to reverse the gene expression signatures of *Setd2*-deficient leukemia. Unlike the conventional approach of selecting candidate drugs based on sRGES, we employed drug enrichment analysis to prioritize drug classes significantly enriched at the top of the prediction. This strategy proved highly effective, as demonstrated by the enrichment of G9a-i, which sensitized *Setd2*-mutant leukemic cells (Figure 5A). While compounds with a stronger reversal of the disease signature were initially hypothesized to be more effective [18], our findings revealed that G9a-i and class I HDAC-i specifically targeted the MYC signature compared with insensitive drugs. In contrast, BET-i, which broadly reversed the disease signature, exhibited severe toxicity, consistent with clinical observations. Supporting our results, Chen et al. demonstrated that effective drugs reversed only 1/10 of the overall disease genes compared to ineffective drugs [18]. These findings highlight the importance of combining experimental validation with computational predictions, or developing new methods to identify critical disease genes for the successful discovery of effective therapeutics.

G9a-i have demonstrated efficacy in various tumors, particularly those with high G9a expression. However, in our study, we found no difference in G9a expression or H3K9me2 levels between *Setd2-*mutant and wild-type leukemic cells. Notably, under G9a inhibition, *Setd2*-mutant cells exhibited a more pronounced repression of the Myc signature compared to wild-type cells. Our previous research identified a critical role for *Setd2* in regulating the quiescence and differentiation of HSPCs by restricting the NSD/SEC-mediated RNA polymerase II elongation on a subset of target genes, including *Myc* [11]. Here, we further confirmed that MYC is essential for the survival of *SETD2*-deficient cells and can be effectively targeted by G9a inhibition. Although MYC is regulated by numerous mechanisms, our study suggests that the H3K9me2/*MIR100HG*/let-7a-2 likely plays a role in suppressing MYC in *SETD2*-deficient cells. While H3K9me2 levels at the *MIR100HG* locus decreased and *MIR100HG* was upregulated in both *SETD2*-deficient and wild-type cells following G9a inhibition, only let-7a-2 was significantly upregulated in *SETD2*-deficient cells. Importantly, let-7a (including let-7a-2) has been shown to directly repress MYC [22,23], which may explain the enhanced MYC repression observed in *SETD2*-deficient cells.

Besides G9a-i, our study also provides insights into the precision medicine application of other drugs in leukemia. *SETD2* mutations are highly prevalent in MLL-r leukemia, and for this study, we utilized an MLL-fusion background. Menin-MLL-i and LSD1-i have entered clinical trials for MLL-r leukemia [31–34]. During compound library screening, we found that both inhibitors showed similar levels of inhibition in *Setd2-*mutant cells and wild-type *Mll-Af9* cells, suggesting that *Setd2* mutation does not affect their efficacy. However, we observed that they consistently inhibited the same proportion of cells across different concentrations (Figure 2B; Table S3), which aligns with the resistance challenges currently observed in clinical settings. Additionally, we discovered that PRMT5-i exhibited resistance in *Setd2-*mutant leukemic cells (Figure S5E; Table S3). Although PRMT5-i have shown promise in treating MLL-r leukemia [36,37], our results suggest that additional mutations, such as those in *SETD2*, should be considered for their potential impact on PRMT5-i efficacy. Further investigation into their functional overlap, such as histone methylation and alternative splicing, is warranted. Furthermore, H3K36me3, mediated by SETD2, interacts with H3K27me3 and DNMTs; however, our results indicate that these interactions do not play a specific role in inhibiting *SETD2*-mutant leukemia.

In conclusion, our study provides new insights into the therapeutic potential for *SETD2-*mutant AML and sheds light on enhancing computational prediction strategies.

## Materials and methods

### Cell lines and culture conditions

The leukemic mouse cell lines and c-kit^+^ HSPCs used in this study were derived from *Mll-Af9/Setd2*^WT/WT^ and *Mll-Af9/Setd2*^F2478L/WT^ mice, as well as wild-type littermates, as previously described by Dong et al. [6]. The cells isolated from these mice have been described and characterized in the same study, and the reduction of H3K36me3 in *Setd2*-mutant cells was confirmed in this study (Figure S6E). *Mll-Af9* and *Mll-Af9/Setd2*^F2478L/WT^ cells were cultured in Iscove’s Modified Dulbecco’s Medium (IMDM; Catalog No. 12440053, Thermo Fisher Scientific, Waltham, MA) supplemented with 10% fetal bovine serum (FBS; Catalog No. 10091148, Thermo Fisher Scientific), 10 ng/ml murine interleukin-3 (IL-3; Catalog No. 213-13, PeproTech, Cranbury NJ), 10 ng/ml murine interleukin-6 (IL-6; Catalog No. 216-16, PeproTech), 10 ng/ml murine stem cell factor (SCF; Catalog No. 250-03, PeproTech), and 10 ng/ml murine granulocyte–macrophage colony-stimulating factor (GM-CSF; Catalog No. 315-03, PeproTech). c-kit^+^ HSPCs were cultured in IMDM supplemented with 10% FBS, 10 ng/ml murine IL-3, 10 ng/ml murine IL-6, and 50 ng/ml murine SCF.

Human THP-1 and KOCL48 cells were cultured in RPMI 1640 medium (Catalog No. C11875500BT, Thermo Fisher Scientific) supplemented with 10% FBS. 293T cells were cultured in Dulbecco’s Modified Eagle’s medium (Catalog No. C11960500BT, Thermo Fisher Scientific) supplemented with 10% FBS. Freshly thawed primary patient leukemic cells were cultured in StemSpan SFEM II (Catalog No. 02605, STEMCELL Technologies, Vancouver, Canada) supplemented with 10 ng/ml human fms-like tyrosine kinase 3 (FLT3; Catalog No. AF-300-19-100, PeproTech), 10 ng/ml human IL-3 (Catalog No. 200-03-100, PeproTech), and 10 ng/ml human SCF (Catalog No. 300-07-100, PeproTech) prior to colony-forming assays.

### Primary patient cells

The primary patient cells were obtained from the peripheral blood or bone marrow of leukemia patients in the Department of Hematology, Beijing Chao-Yang Hospital, Capital Medical University, Beijing, China. Detailed clinical information is provided in Table S9. To verify *SETD2* LOF mutations, we analyzed H3K36me3 levels by Western blot but did not observe significant differences between *SETD2*-mutant and wild-type cells (data not shown), potentially due to patient heterogeneity. However, *MYC* expression was relatively higher in *SETD2*-mutant cells compared to wild-type controls (Figure S11D), supporting the presence of a MYC signature and their increased sensitivity to G9a-i.

### Disease signature establishment

To establish the *Setd2-*mutant leukemia signature, RNA-seq was performed on *Mll-Af9/Setd2*^F2478L/WT^ and *Mll-Af9* cells to identify DEGs. Total RNA was extracted from *Mll-Af9/Setd2*^F2478L/WT^ and *Mll-Af9* cells using TRIzol reagent (Catalog No. 10296028, Thermo Fisher Scientific). RNA-seq libraries were prepared using the KAPA RNA HyperPrep Kit with RiboErase (Catalog No. KK8560, Roche, Basel, Switzerland) following the manufacturer’s instructions and sequenced on the Illumina NovaSeq platform (Illumina, San Diego, CA) with 150 bp paired-end reads. Raw sequencing reads were processed using FastQC for quality control and Trimmomatic (v0.36) [43] for trimming, and then aligned to the mouse reference genome (mm10) using HISAT2 [44]. DEGs were identified using DESeq2 [45] with a threshold of false discovery rate (FDR) ≤ 0.05 and |log_2_ FC| ≥ 0.5. For the *Setd2* knockdown leukemia signature, DEGs (FDR ≤ 0.05 and FC ≥ 1.2 or ≤ 0.8) from *Mll-Af9/*sh*Setd2 versus Mll-Af9* cells, obtained from our previously study [12], were used.

### Computational prediction of candidate drugs

The computational algorithm for drug prediction, developed by Sirota et al. [16] and adapted from Chen et al. [18], was applied to the *Setd2-*deficient leukemia signatures using the publicly available LINCS L1000 database (https://lincsportal.ccs.miami.edu) [17]. The algorithm relies on two primary inputs: (1) a list of upregulated and downregulated genes in the disease, and (2) drug signatures from L1000, which consist of rank-ordered FC of each gene after drug treatment under various conditions, including duration time, concentration, and cell lines. For this study, drug signatures from hematopoietic cell lines were exclusively used due to their higher correlation with our model compared to other lineages in the L1000 database (data not shown). Mouse gene symbols in the leukemia signatures were converted to their human homologs to ensure compatibility with the database. A Kolmogorov-Smirnov test was employed to compute the positions of the disease gene set within each drug signature and assign a RGES, reflecting the extent to which a drug under specific conditions reverses the disease signature. A sRGES, normalized across different conditions, was calculated for each drug. For drug enrichment analysis, drugs sharing the same MOA were grouped into drug classes, and Gene Set Variation Analysis was performed to obtain an enrichment score for each class. The sRGES of drugs within each class was compared to all others using one-tailed *t*-test to assess statistical significance. To visualize drug signatures against the disease gene set, the mean gene FC across different conditions for each drug was used for ranking and visualization. Genes reversed by candidate drug classes were identified by selecting those reversed in more than 60% of the profiles within a drug class.

### Cell viability assays for epigenetic compound library screening, dose-response curves, and cell proliferation assessment

A customized library containing 215 epigenetic inhibitors was constructed based on the MedChemExpress epigenetic inhibitor compound library (Catalog No. HY-L005, MedChemExpress, Monmouth Junction, NJ). These inhibitors target epigenetic writers, erasers, and readers. *Mll-Af9* and *Mll-Af9/Setd2*^F2478L/WT^ cells were seeded at a density of 1000 cells per well in 96-well plates and treated with the inhibitor library at five concentrations (0.05, 0.2, 1, 2, and 5 μM), along with DMSO and no-vehicle controls. After 5 days, cell viability was assessed using the CellTiter-Blue Cell Viability Assay (Catalog No. G8081, Promega, Madison, WI). Absorbance values were normalized within plates (DMSO-treated cells) and across plates (empty wells). Data visualization and clustering were performed using the R package.

For dose-response curves, detailed concentrations and treatment times for each drug are listed in Table S3. For Ara-C treatment, 10,000 cells per well were seeded, while 1000 cells per well were used for other drugs. Cell viability was measured at the end of the treatment as described above.

To assess proliferation changes in *Mll-Af9* and *Mll-Af9/Setd2*^F2478L/WT^ cells after *G9a* or *Myc* knockdown, 1000 cells per well of each genotype were seeded in 96-well plates. Cell viability was assessed daily for 4 or 5 consecutive days using the same method as described above.

All data were analyzed and plotted using GraphPad Prism (v8). Data were presented as mean ± standard error of the mean (SEM; *n* = 3). Statistical significance was calculated using two-way ANOVA.

### Flow cytometry analyses of apoptosis, cell cycle, and cell differentiation

*Mll-Af9* and *Mll-Af9/Setd2*^F2478L/WT^ cells were treated with UNC0642 (Catalog No. HY-13980, MedChemExpress) at concentrations of 2 µM and 4 µM, or with a DMSO control, at a density of 20,000 cells per well in 6-well plates for 72 h. For apoptosis assessment, cells were stained with the Annexin V-FITC/7-AAD apoptosis kit (Catalog No. 640922, BioLegend, San Diego, CA) following the manufacturer’s instructions. For cell cycle analysis, cells were incubated with EdU for 1 h before harvest, followed by fixation and staining using the Click-iT EdU Alexa Fluor 647 kit (Catalog No. C10424, Thermo Fisher Scientific) according to the manufacturer’s instructions. Cell differentiation was assessed by staining cells with CD11b-FITC (Catalog No. 11-0118-42, Thermo Fisher Scientific) after Fc receptor blocking (Catalog No. 101301, BioLegend). Flow cytometry analyses were performed on BD FACS Aria or MoFlo XDP flow cytometers, and data were analyzed using FlowJo software. All statistical analyses were performed using GraphPad Prism (v8), and data are presented as mean ± SEM (*n* = 3). Statistical significance was calculated using two-way ANOVA.

### Colony-forming assay

*Mll-Af9* and *Mll-Af9/Setd2*^F2478L/WT^ cells were treated with UNC0642 in the “Flow cytometry analyses” section. Subsequently, 10,000 cells per condition were plated in 1 ml MethoCult GF M3434 methylcellulose-based medium (Catalog No. 03434, STEMCELL Technologies). Colonies were scored and re-plated every 5 days.

For primary patient leukemic cells, 10,000 cells/ml were seeded in MethoCult H4435 Enriched (Catalog No. H4435, STEMCELL Technologies) with 2 µM UNC0642 or DMSO control. After 2 weeks, colonies were counted, harvested, and used for total cell number counting and RNA extraction.

All statistical analyses were performed using GraphPad Prism (v8), and data are presented as mean ± SEM (*n* = 3 or *n* = 4). Statistical significance was calculated using two-way ANOVA.

### Cell transfection and lentivirus transduction

To produce lentiviruses expressing shRNA, 293T cells were transfected with a combination of a shRNA expression lentiviral vector and packaging plasmids (pMD2.G and psPAX2) using FuGENE HD (Catalog No. E2311, Promega). After 48 h, lentiviral supernatants were harvested, filtered through a 0.45-µm filter, and immediately used to infect target cells. Successfully infected cells were selected with puromycin (Catalog No. A1113803, Thermo Fisher Scientific) at a final concentration of 1 µg/ml or by flow cytometry. The sequences of the shRNAs used in this study are listed in Table S8.

### Reverse transcription quantitative PCR

Total RNA was extracted using TRIzol reagent (Catalog No. 15596018, Thermo Fisher Scientific). cDNA synthesis was performed using distinct protocols for different RNA species: (1) mRNAs and lncRNAs were reverse transcribed using the Reverse Transcription System (Catalog No. A3500, Promega); (2) miRNAs were reverse transcribed using stem-loop-specific primers to ensure accurate transcription of small RNAs. Quantitative PCR (qPCR) was performed using the KAPA SYBR FAST kit (Catalog No. KK4601, Roche). The primers used in this study are listed in Table S8.

### Immunoblotting

Cell extracts were prepared by lysing cells in sodium dodecyl sulfate (SDS) sample buffer containing 10 mM NaF, 0.2 mM Na_3_VO_4_, 10 mM glycerophosphate, 1 mM PMSF, 2.5 mM DTT, and a protease inhibitor cocktail, followed by 30 s of sonication. Samples were boiled at 95°C for 5 min, separated by SDS-polyacrylamide gel electrophoresis, and electro-transferred to PVDF membranes. The primary antibodies used in this study included: anti-β-Actin (Catalog No. ab49900, Abcam, Cambridge, UK), anti-H3K36me3 (Catalog No. ab9050, Abcam), anti-H3K9me2 (Catalog No. ab1220, Abcam), anti-H3 (Catalog No. ab1791, Abcam), anti-SETD2 (Catalog No. ab184190, Abcam; Catalog No. A3194, Abclone, Wuhan, China), anti-G9a (Catalog No. ab185050, Abcam), anti-MYC (Catalog No. 13987S, Cell Signaling Technology, Danvers, MA), anti-LC3B (Catalog No. ab1520, Abcam), and anti-GAPDH (Catalog No. ab9485, Abcam). Secondary antibodies were horseradish peroxidase-conjugated antibodies against goat, rabbit, or mouse immunoglobulins. SuperSignal West Dura and Femto Chemiluminescent Substrate (Catalog Nos. 34075 and 34095, Thermo Fisher Scientific) were used for enhanced chemiluminescence detection. Band intensities were quantified using ImageJ software.

### RNA-seq and ChIP-seq

*Mll-Af9* and *Mll-Af9/Setd2*^F2478L/WT^ cells treated with 2 µM UNC0642 or DMSO control for 72 h were used for RNA-seq and ChIP-seq. RNA extraction and sequencing were performed as described in the “Disease signature establishment” section, and DEGs were filtered using a FDR threshold of ≤ 0.05.

ChIP was performed using in-house reagents. Cells were crosslinked with 1% formaldehyde for 10 min at room temperature, and the reaction was quenched with 0.125 M glycine. Chromatin fragments were precleared and immunoprecipitated using ChIP-grade protein G magnetic beads (Catalog No. 9006S, Cell Signaling Technology) coupled with anti-H3K9me2 antibody (Catalog No. ab1220, Abcam) or immunoglobulin G (Catalog No. ab171870, Abcam) as a control. After reverse crosslinking, H3K9me2-ChIP and input DNA fragments were used to construct DNA libraries using the NEBNext DNA Library Prep Kit (Catalog No. E7103, NEB, Ipswich, MA) according to the protocol. Libraries passing quality control were sequenced on the Illumina NovaSeq platform with 150 bp paired-end reads. Raw sequencing reads were processed using FastQC for quality control and Trimmomatic (v0.36) [43] for adapter trimming, followed by alignment to the mouse reference genome (mm10) using Bowtie2 [46]. EPIC2 [47] was used for peak calling with a bin size of 5000 bp relative to the input control. Differential peak analysis between experimental groups was performed using DiffBind. Gene and pathway annotations were generated using ChIPseeker. Peaks were visualized using Integrative Genomics Viewer [48]. Primers for ChIP-qPCR are listed in Table S8.

### Gene enrichment analyses

Enrichment analyses of DEGs were performed and visualized using various bioinformatics tools with the default parameters. Metascape (v3.5) [49] was utilized to evaluate the biological functions of the *Setd2-*deficient leukemia signatures, genes reversed by candidate drug classes in L1000, and DEGs following UNC0642 treatment in *Mll-Af9* and *Mll-Af9/Setd2*^F2478L/WT^ cells. Cytoscape (v3.10.2) [50] was employed to visualize the enriched functional networks of genes reversed by candidate drug classes in L1000. IPA [51] was used to assess the canonical biological pathways and upstream regulators of DEGs. GSEA (v4.0.3) [52] was used to evaluate the activities of MYC-related and lysosome/autophagy-related gene sets in *Mll-Af9* and *Mll-Af9/Setd2*^F2478L/WT^ cells under UNC0642 treatment compared to DMSO controls. Gene sets were downloaded from the Molecular Signatures Database (MSigDB; v7.0) of the Broad Institute.

### Computational prediction and phenotypic outcome correlation analysis and reversal gene identification

To establish the correlation between computational predictions and phenotypic outcomes, overlapping drugs were selected. For drugs with IC50 values, the FC of IC50 between *Setd2-*mutant and wild-type cells was used to represent the drug response. For drugs without IC50 values, the mean FC of cell viability between the two cell types at five concentrations in the compound library screening was used to represent the drug response. G9a-i and class I HDAC-i were categorized as sensitive to *Setd2*-deficient leukemia, while ATM/ATR-i and BTK-i were categorized as insensitive. BET-i were excluded due to their potential over-reversal effects. All expression profiles of these drugs in the L1000 database were used for the analysis. Reversal genes were defined as those ranked lower for upregulated genes and higher for downregulated genes in the *Setd2*-deficient leukemia-sensitive group compared to the insensitive group. A one-sided Mann-Whitney-Wilcoxon test was used to assess the significance of rank differences between the two groups. Genes with FDR ≤ 0.25 and reversed in at least 60% of profiles with G9a-i or class I HDAC-i were considered reversal genes.

## Ethical statement

The use of primary patient cells, as well as the review of all pertinent patient records, was conducted in accordance with the ethical standards of the Declaration of Helsinki and was approved by the Research Ethics Committee of Beijing Chao-Yang Hospital, Capital Medical University, China (Approval No. 2024-6-27-6). Informed consent was obtained from all patients.

## Supplementary Material

qzaf035_Supplementary_Data

## Data Availability

The raw sequence data reported in this study have been deposited in the Genome Sequence Archive [53] at the National Genomics Data Center (NGDC), China National Center for Bioinformation (CNCB) (GSA: CRA016934 and CRA023496), and are publicly accessible at https://ngdc.cncb.ac.cn/gsa.

## CRediT author statement

**Ya Zhang:** Formal analysis, Experiment performance, Investigation, Validation, Visualization, Writing – original draft. **Mengfang Xia:** Experiment performance, Investigation, Validation, Visualization, Writing – original draft. **Zhenyi Yi:** Experiment performance, Investigation. **Pinpin Sui:** Formal analysis. **Xudong He:** Formal analysis. **Liping Wang:** Experiment performance. **Qiyi Chen:** Experiment performance. **Hong-Hu Zhu:** Resources. **Gang Huang:** Conceptualization, Project administration, Supervision, Writing – review & editing. **Qian-Fei Wang:** Conceptualization, Funding acquisition, Project administration, Supervision, Writing – review & editing. All authors have read and approved the final manuscript.
