# Supplementary material for: Integrated Computational and Functional Screening Identifies G9a Inhibitors for SETD2-mutant Leukemia
Source: Genomics Proteomics Bioinformatics. 2025 Apr 29;23(2):qzaf035. doi: 10.1093/gpbjnl/qzaf035 (PMC12373973; doi:10.1093/gpbjnl/qzaf035)
Supplement: qzaf035_Supplementary_Data [file qzaf035_supplementary_data.zip › qzaf035_Supplementary_Data.zip!/Figure S1.pdf]

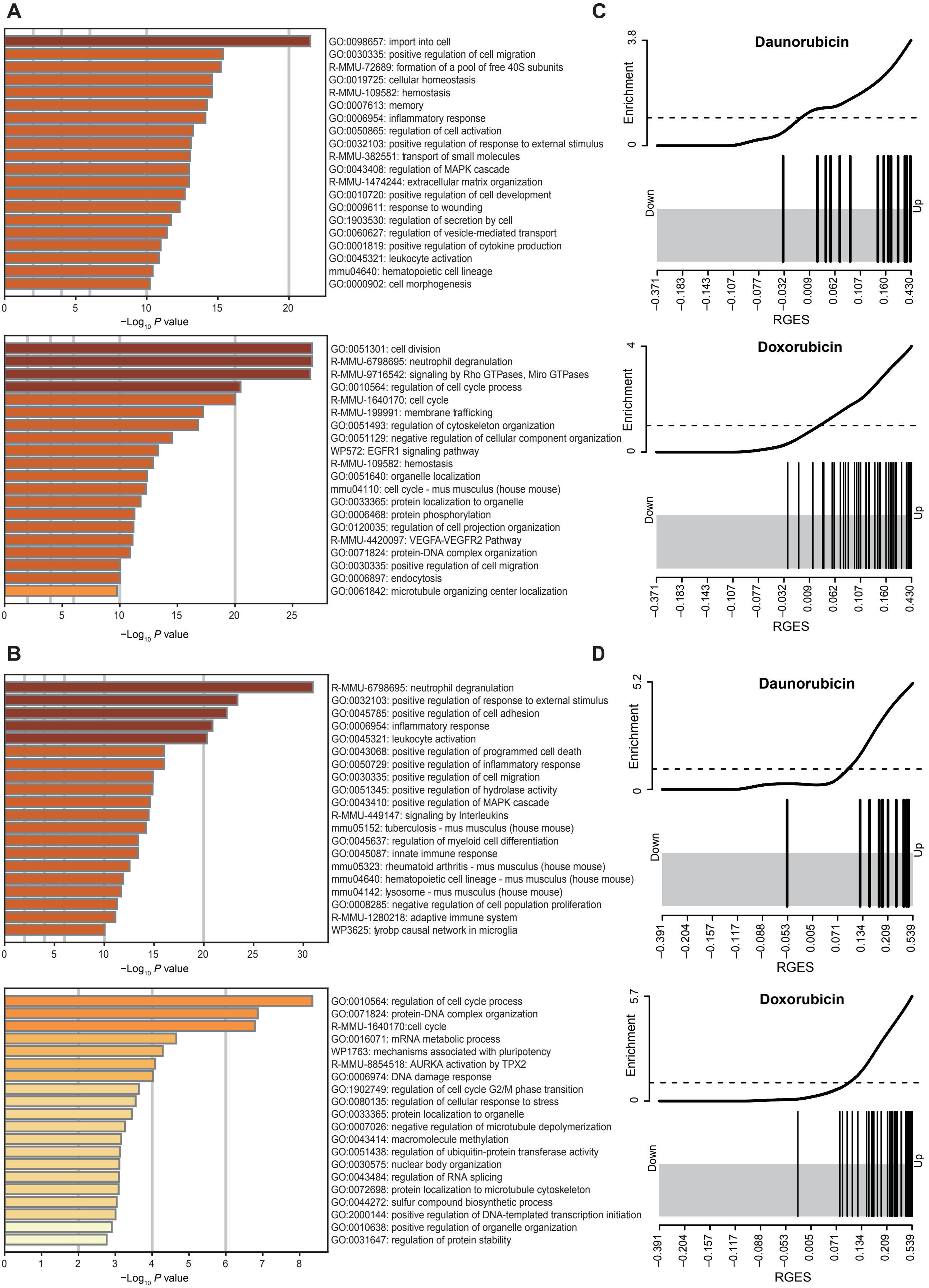
A
GO:0098657: import into cell
GO:0030335: positive regulation of cell migration
R-MMU-72689: formation of a pool of free 40S subunits
GO:0019725: cellular homeostasis
R-MMU-109582: hemostasis
GO:0007613: memory
GO:0006954: inflammatory response
GO:0050865: regulation of cell activation
GO:0032103: positive regulation of response to external stimulus
R-MMU-382551: transport of small molecules
GO:0043408: regulation of MAPK cascade
R-MMU-1474244: extracellular matrix organization
GO:0010720: positive regulation of cell development
GO:0009611: response to wounding
GO:1903530: regulation of secretion by cell
GO:0060627: regulation of vesicle-mediated transport
GO:0001819: positive regulation of cytokine production
GO:0045321: leukocyte activation
mmu04640: hematopoietic cell lineage
GO:0000902: cell morphogenesis
0 5 10 15 20
−Log10 P value
GO:0051301: cell division
R-MMU-6798695: neutrophil degranulation
R-MMU-9716542: signaling by Rho GTPases, Miro GTPases
GO:0010564: regulation of cell cycle process
R-MMU-1640170: cell cycle
R-MMU-199991: membrane trafficking
GO:0051493: regulation of cytoskeleton organization
GO:0051129: negative regulation of cellular component organization
WP572: EGFR1 signaling pathway
R-MMU-109582: hemostasis
GO:0051640: organelle localization
mmu04110: cell cycle - mus musculus (house mouse)
GO:0033365: protein localization to organelle
GO:0006468: protein phosphorylation
GO:0120035: regulation of cell projection organization
R-MMU-4420097: VEGFA-VEGFR2 Pathway
GO:0071824: protein-DNA complex organization
GO:0030335: positive regulation of cell migration
GO:0006897: endocytosis
GO:0061842: microtubule organizing center localization
0 5 10 15 20 25
−Log10 P value
C
Daunorubicin
Enrichment
3.8
0
Down
Up
−0.371 −0.183 −0.143 −0.107 −0.077 −0.032 0.009 0.062 0.107 0.160 0.430
RGES
Doxorubicin
Enrichment
4
0
Down
Up
−0.371 −0.183 −0.143 −0.107 −0.077 −0.032 0.009 0.062 0.107 0.160 0.430
RGES
B
R-MMU-6798695: neutrophil degranulation
GO:0032103: positive regulation of response to external stimulus
GO:0045785: positive regulation of cell adhesion
GO:0006954: inflammatory response
GO:0045321: leukocyte activation
GO:0043068: positive regulation of programmed cell death
GO:0050729: positive regulation of inflammatory response
GO:0030335: positive regulation of cell migration
GO:0051345: positive regulation of hydrolase activity
GO:0043410: positive regulation of MAPK cascade
R-MMU-449147: signaling by Interleukins
mmu05152: tuberculosis - mus musculus (house mouse)
GO:0045637: regulation of myeloid cell differentiation
GO:0045087: innate immune response
mmu05323: rheumatoid arthritis - mus musculus (house mouse)
mmu04640: hematopoietic cell lineage - mus musculus (house mouse)
mmu04142: lysosome - mus musculus (house mouse)
GO:0008285: negative regulation of cell population proliferation
R-MMU-1280218: adaptive immune system
WP3625: tyrobp causal network in microglia
0 5 10 15 20 25 30
−Log10 P value
GO:0010564: regulation of cell cycle process
GO:0071824: protein-DNA complex organization
R-MMU-1640170:cell cycle
GO:0016071: mRNA metabolic process
WP1763: mechanisms associated with pluripotency
R-MMU-8854518: AURKA activation by TPX2
GO:0006974: DNA damage response
GO:1902749: regulation of cell cycle G2/M phase transition
GO:0080135: regulation of cellular response to stress
GO:0033365: protein localization to organelle
GO:0007026: negative regulation of microtubule depolymerization
GO:0043414: macromolecule methylation
GO:0051438: regulation of ubiquitin-protein transferase activity
GO:0030575: nuclear body organization
GO:0043484: regulation of RNA splicing
GO:0072698: protein localization to microtubule cytoskeleton
GO:0044272: sulfur compound biosynthetic process
GO:2000144: positive regulation of DNA-templated transcription initiation
GO:0010638: positive regulation of organelle organization
GO:0031647: regulation of protein stability
0 1 2 3 4 5 6 7 8
−Log10 P value
D
Daunorubicin
Enrichment
5.2
0
Down
Up
−0.391 −0.204 −0.157 −0.117 −0.088 −0.053 0.005 0.071 0.134 0.209 0.539
RGES
Doxorubicin
Enrichment
5.7
0
Down
Up
−0.391 −0.204 −0.157 −0.117 −0.088 −0.053 0.005 0.071 0.134 0.209 0.539
RGES
