# Supplementary material for: Integrated Computational and Functional Screening Identifies G9a Inhibitors for SETD2-mutant Leukemia
Source: Genomics Proteomics Bioinformatics. 2025 Apr 29;23(2):qzaf035. doi: 10.1093/gpbjnl/qzaf035 (PMC12373973; doi:10.1093/gpbjnl/qzaf035)
Supplement: qzaf035_Supplementary_Data [file qzaf035_supplementary_data.zip › qzaf035_Supplementary_Data.zip!/Figure S2.pdf]

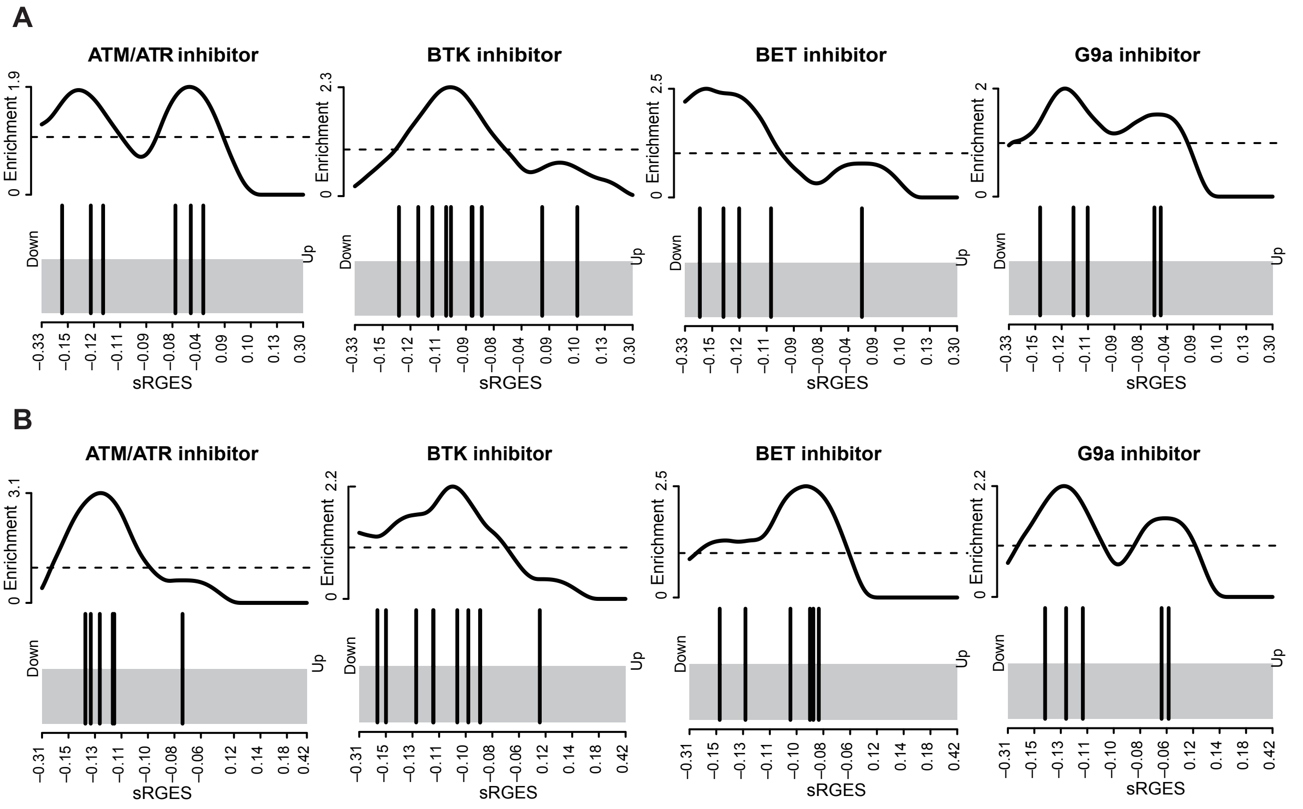
A
ATM/ATR inhibitor
Enrichment
0
1.9
Down
Up
−0.33 −0.15 −0.12 −0.11 −0.09 −0.08 −0.04 0.09 0.10 0.13 0.30
sRGES
BTK inhibitor
Enrichment
0
2.3
Down
Up
−0.33 −0.15 −0.12 −0.11 −0.09 −0.08 −0.04 0.09 0.10 0.13 0.30
sRGES
BET inhibitor
Enrichment
0
2.5
Down
Up
−0.33 −0.15 −0.12 −0.11 −0.09 −0.08 −0.04 0.09 0.10 0.13 0.30
sRGES
G9a inhibitor
Enrichment
0
2
Down
Up
−0.33 −0.15 −0.12 −0.11 −0.09 −0.08 −0.04 0.09 0.10 0.13 0.30
sRGES
B
ATM/ATR inhibitor
Enrichment
0
3.1
Down
Up
−0.31 −0.15 −0.13 −0.11 −0.10 −0.08 −0.06 0.12 0.14 0.18 0.42
sRGES
BTK inhibitor
Enrichment
0
2.2
Down
Up
−0.31 −0.15 −0.13 −0.11 −0.10 −0.08 −0.06 0.12 0.14 0.18 0.42
sRGES
BET inhibitor
Enrichment
0
2.5
Down
Up
−0.31 −0.15 −0.13 −0.11 −0.10 −0.08 −0.06 0.12 0.14 0.18 0.42
sRGES
G9a inhibitor
Enrichment
0
2.2
Down
Up
−0.31 −0.15 −0.13 −0.11 −0.10 −0.08 −0.06 0.12 0.14 0.18 0.42
sRGES
