# Supplementary material for: Integrated Computational and Functional Screening Identifies G9a Inhibitors for SETD2-mutant Leukemia
Source: Genomics Proteomics Bioinformatics. 2025 Apr 29;23(2):qzaf035. doi: 10.1093/gpbjnl/qzaf035 (PMC12373973; doi:10.1093/gpbjnl/qzaf035)
Supplement: qzaf035_Supplementary_Data [file qzaf035_supplementary_data.zip › qzaf035_Supplementary_Data.zip!/Figure S3.pdf]

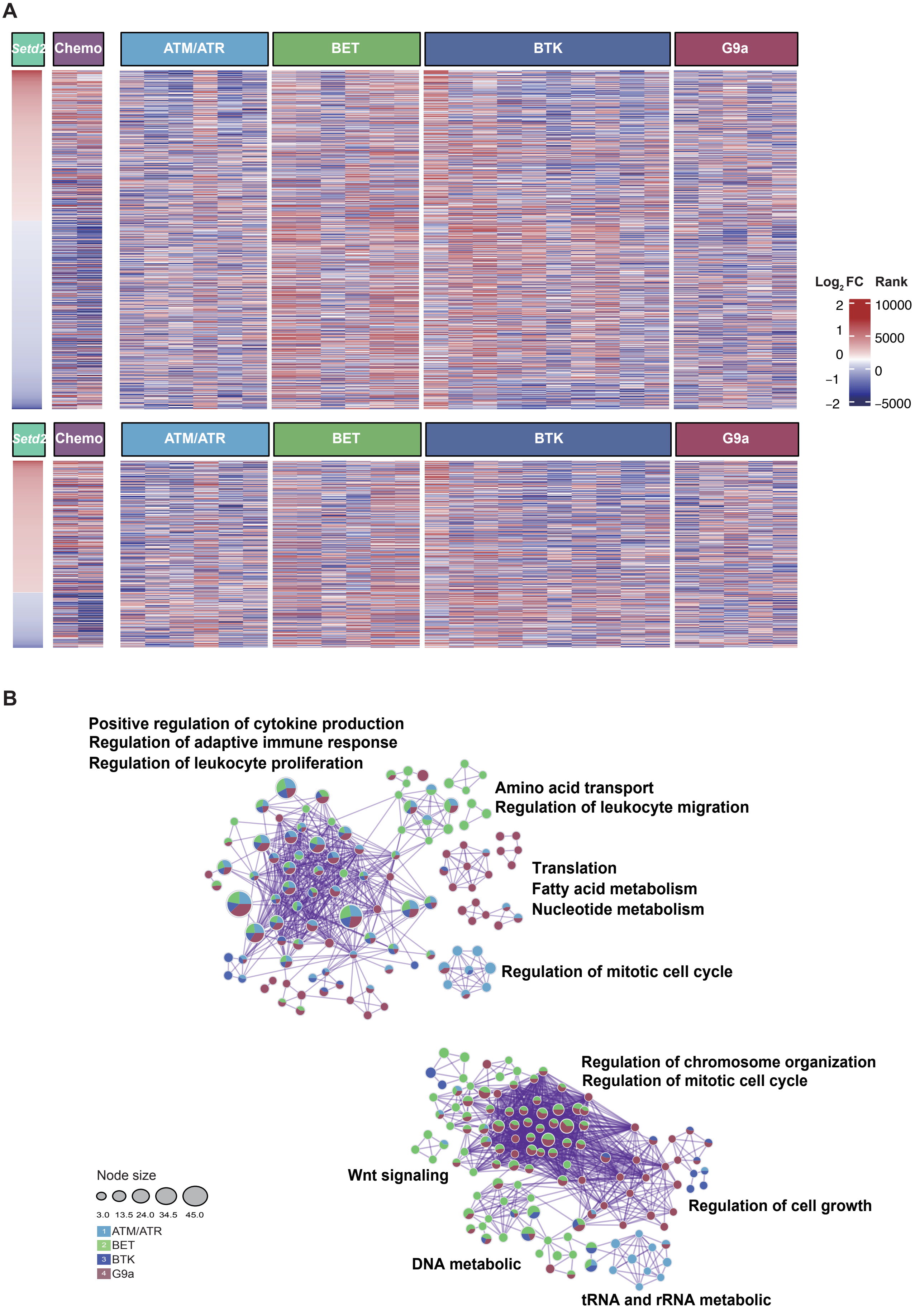
A
Setd2
Chemo
ATM/ATR
BET
BTK
G9a
Log2FC
Rank
2
1
0
−1
−2
10000
5000
0
−5000
Setd2
Chemo
ATM/ATR
BET
BTK
G9a
B
Positive regulation of cytokine production
Regulation of adaptive immune response
Regulation of leukocyte proliferation
Amino acid transport
Regulation of leukocyte migration
Translation
Fatty acid metabolism
Nucleotide metabolism
Regulation of mitotic cell cycle
Regulation of chromosome organization
Regulation of mitotic cell cycle
Wnt signaling
Regulation of cell growth
DNA metabolic
tRNA and rRNA metabolic
Node size
3.0 13.5 24.0 34.5 45.0
1 ATM/ATR
2 BET
3 BTK
4 G9a
