# Supplementary material for: Integrated Computational and Functional Screening Identifies G9a Inhibitors for SETD2-mutant Leukemia
Source: Genomics Proteomics Bioinformatics. 2025 Apr 29;23(2):qzaf035. doi: 10.1093/gpbjnl/qzaf035 (PMC12373973; doi:10.1093/gpbjnl/qzaf035)
Supplement: qzaf035_Supplementary_Data [file qzaf035_supplementary_data.zip › qzaf035_Supplementary_Data.zip!/Figure S4.pdf]

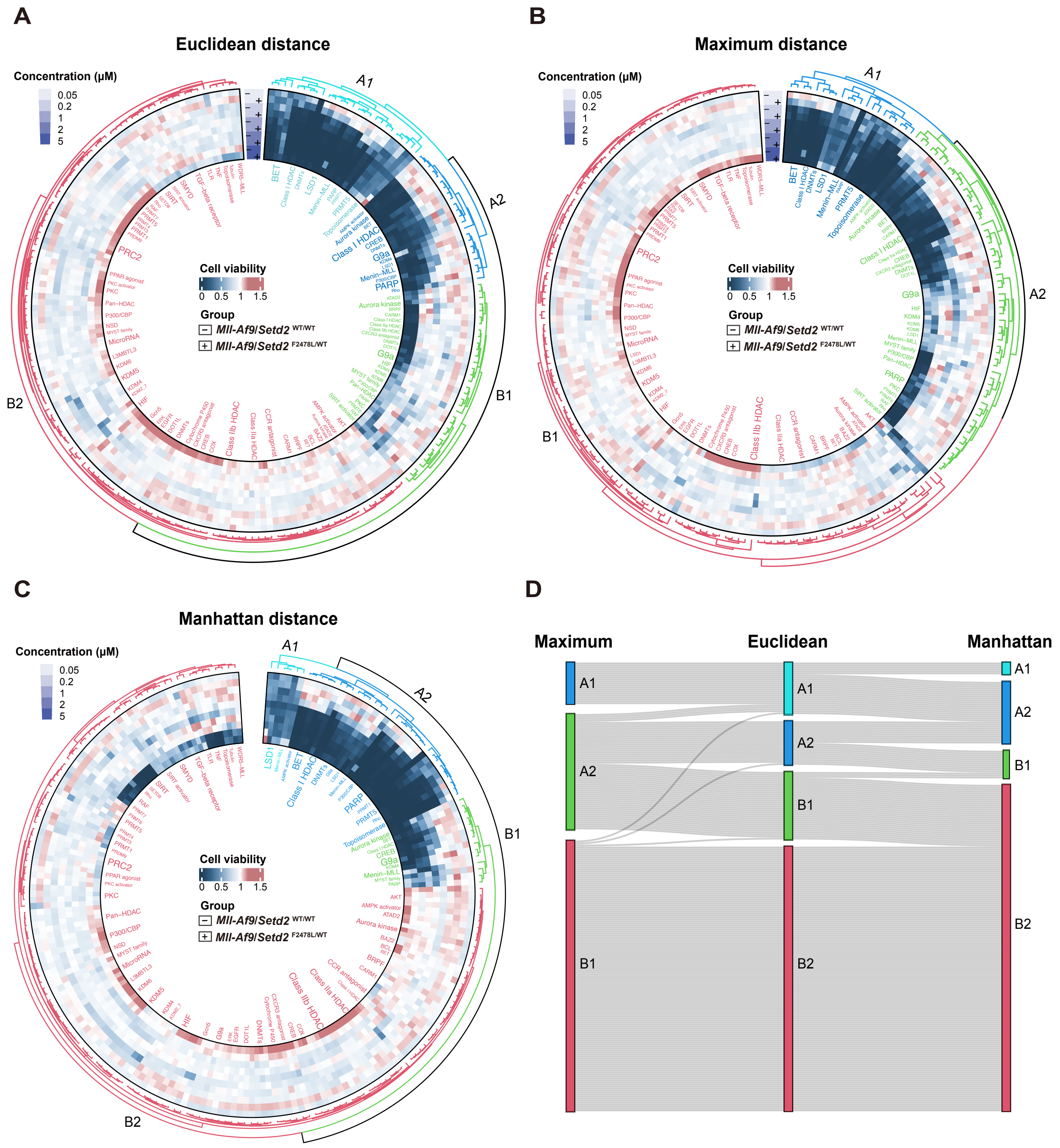

A
Euclidean distance
Concentration (μM)
0.05
0.2
1
2
5
A1
A2
B1
B2
Cell viability
0
0.5
1
1.5
Group
Mll-Af9/Setd2 WT/WT
Mll-Af9/Setd2 F2478L/WT
B
Maximum distance
Concentration (μM)
0.05
0.2
1
2
5
A1
A2
B1
Cell viability
0
0.5
1
1.5
Group
Mll-Af9/Setd2 WT/WT
Mll-Af9/Setd2 F2478L/WT
C
Manhattan distance
Concentration (μM)
0.05
0.2
1
2
5
A1
A2
B1
B2
Cell viability
0
0.5
1
1.5
Group
Mll-Af9/Setd2 WT/WT
Mll-Af9/Setd2 F2478L/WT
D
Maximum
Euclidean
Manhattan
A1
A2
B1
A1
A2
B1
B2
A1
A2
B1
B2
