# Supplementary material for: Integrated Computational and Functional Screening Identifies G9a Inhibitors for SETD2-mutant Leukemia
Source: Genomics Proteomics Bioinformatics. 2025 Apr 29;23(2):qzaf035. doi: 10.1093/gpbjnl/qzaf035 (PMC12373973; doi:10.1093/gpbjnl/qzaf035)
Supplement: qzaf035_Supplementary_Data [file qzaf035_supplementary_data.zip › qzaf035_Supplementary_Data.zip!/Figure S5.pdf]

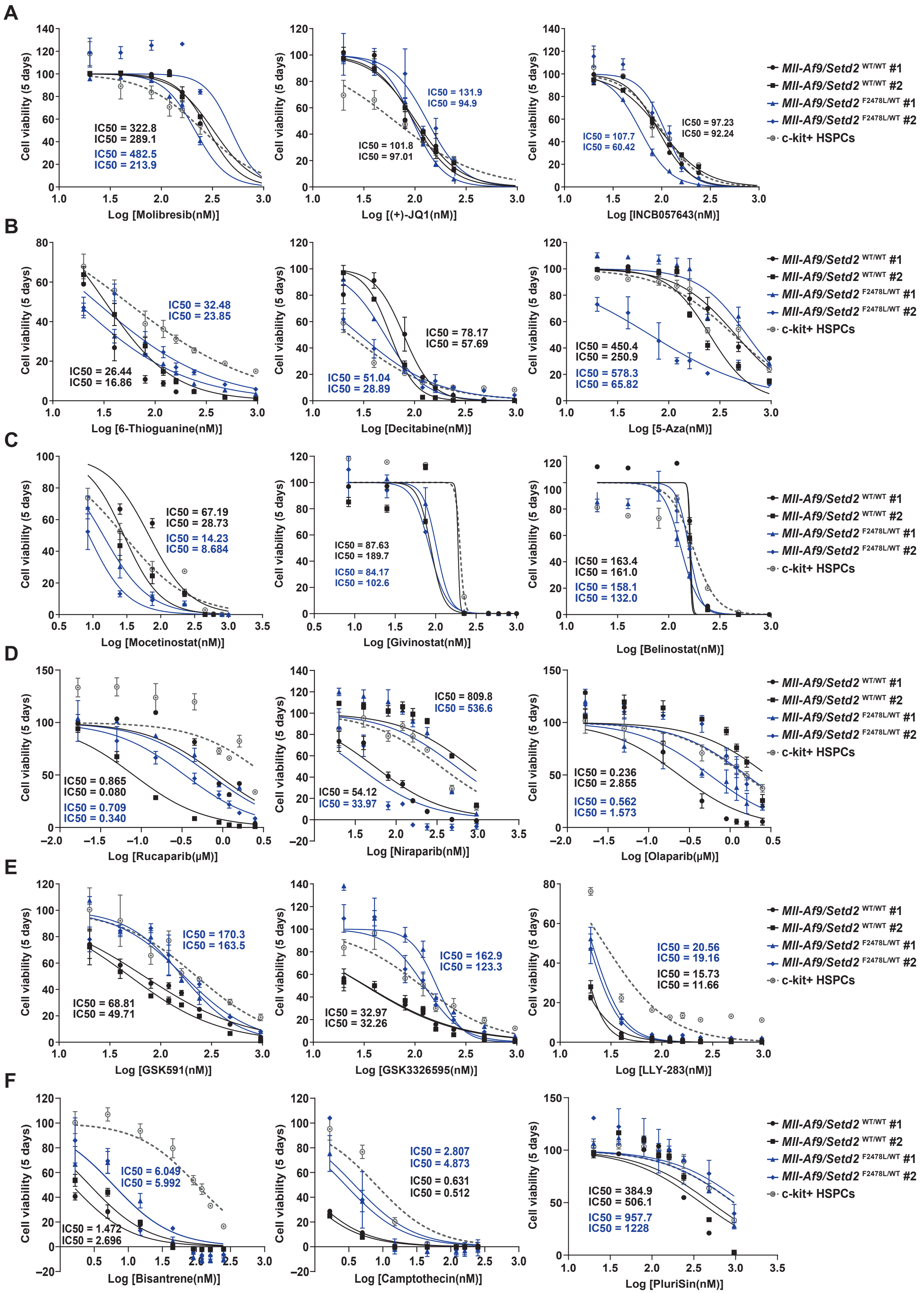
A
Cell viability (5 days)
IC50 = 322.8
IC50 = 289.1
IC50 = 482.5
IC50 = 213.9
Log [Molibresib(nM)]
IC50 = 131.9
IC50 = 94.9
IC50 = 101.8
IC50 = 97.01
Log [(+)-JQ1(nM)]
IC50 = 97.23
IC50 = 92.24
IC50 = 107.7
IC50 = 60.42
Log [INCB057643(nM)]
Mll-Af9/Setd2 WT/WT #1
Mll-Af9/Setd2 WT/WT #2
Mll-Af9/Setd2 F2478L/WT #1
Mll-Af9/Setd2 F2478L/WT #2
c-kit+ HSPCs
B
IC50 = 32.48
IC50 = 23.85
IC50 = 26.44
IC50 = 16.86
Log [6-Thioguanine(nM)]
IC50 = 78.17
IC50 = 57.69
IC50 = 51.04
IC50 = 28.89
Log [Decitabine(nM)]
IC50 = 450.4
IC50 = 250.9
IC50 = 578.3
IC50 = 65.82
Log [5-Aza(nM)]
C
IC50 = 67.19
IC50 = 28.73
IC50 = 14.23
IC50 = 8.684
Log [Mocetinostat(nM)]
IC50 = 87.63
IC50 = 189.7
IC50 = 84.17
IC50 = 102.6
Log [Givinostat(nM)]
IC50 = 163.4
IC50 = 161.0
IC50 = 158.1
IC50 = 132.0
Log [Belinostat(nM)]
D
IC50 = 0.865
IC50 = 0.080
IC50 = 0.709
IC50 = 0.340
Log [Rucaparib(μM)]
IC50 = 809.8
IC50 = 536.6
IC50 = 54.12
IC50 = 33.97
Log [Niraparib(nM)]
IC50 = 0.236
IC50 = 2.855
IC50 = 0.562
IC50 = 1.573
Log [Olaparib(μM)]
E
IC50 = 170.3
IC50 = 163.5
IC50 = 68.81
IC50 = 49.71
Log [GSK591(nM)]
IC50 = 162.9
IC50 = 123.3
IC50 = 32.97
IC50 = 32.26
Log [GSK3326595(nM)]
IC50 = 20.56
IC50 = 19.16
IC50 = 15.73
IC50 = 11.66
Log [LLY-283(nM)]
F
IC50 = 6.049
IC50 = 5.992
IC50 = 1.472
IC50 = 2.696
Log [Bisantrene(nM)]
IC50 = 2.807
IC50 = 4.873
IC50 = 0.631
IC50 = 0.512
Log [Camptothecin(nM)]
IC50 = 384.9
IC50 = 506.1
IC50 = 957.7
IC50 = 1228
Log [PluriSin(nM)]
