# Supplementary material for: Integrated Computational and Functional Screening Identifies G9a Inhibitors for SETD2-mutant Leukemia
Source: Genomics Proteomics Bioinformatics. 2025 Apr 29;23(2):qzaf035. doi: 10.1093/gpbjnl/qzaf035 (PMC12373973; doi:10.1093/gpbjnl/qzaf035)
Supplement: qzaf035_Supplementary_Data [file qzaf035_supplementary_data.zip › qzaf035_Supplementary_Data.zip!/Figure S6.pdf]

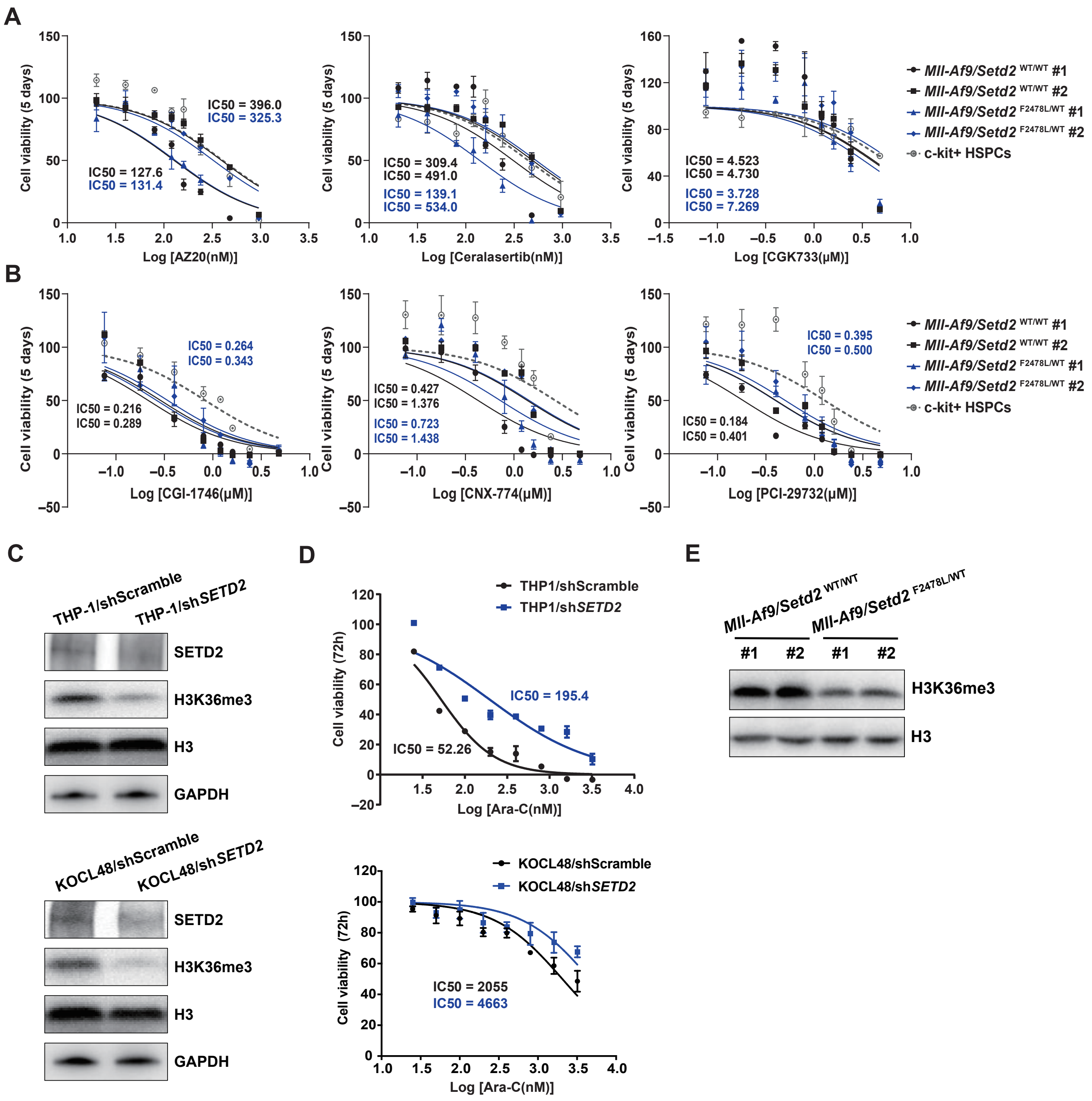

A
IC50 = 396.0
IC50 = 325.3
IC50 = 127.6
IC50 = 131.4
Cell viability (5 days)
Log [AZ20(nM)]
IC50 = 309.4
IC50 = 491.0
IC50 = 139.1
IC50 = 534.0
Log [Ceralasertib(nM)]
IC50 = 4.523
IC50 = 4.730
IC50 = 3.728
IC50 = 7.269
Log [CGK733(μM)]
Mll-Af9/Setd2 WT/WT #1
Mll-Af9/Setd2 WT/WT #2
Mll-Af9/Setd2 F2478L/WT #1
Mll-Af9/Setd2 F2478L/WT #2
c-kit+ HSPCs
B
IC50 = 0.264
IC50 = 0.343
IC50 = 0.216
IC50 = 0.289
Log [CGI-1746(μM)]
IC50 = 0.427
IC50 = 1.376
IC50 = 0.723
IC50 = 1.438
Log [CNX-774(μM)]
IC50 = 0.395
IC50 = 0.500
IC50 = 0.184
IC50 = 0.401
Log [PCI-29732(μM)]
C
THP-1/shScramble
THP-1/shSETD2
SETD2
H3K36me3
H3
GAPDH
KOCL48/shScramble
KOCL48/shSETD2
D
THP1/shScramble
THP1/shSETD2
IC50 = 195.4
IC50 = 52.26
Cell viability (72h)
Log [Ara-C(nM)]
KOCL48/shScramble
KOCL48/shSETD2
IC50 = 2055
IC50 = 4663
E
Mll-Af9/Setd2 WT/WT
Mll-Af9/Setd2 F2478L/WT
#1
#2
H3K36me3
H3
