# Supplementary material for: Integrated Computational and Functional Screening Identifies G9a Inhibitors for SETD2-mutant Leukemia
Source: Genomics Proteomics Bioinformatics. 2025 Apr 29;23(2):qzaf035. doi: 10.1093/gpbjnl/qzaf035 (PMC12373973; doi:10.1093/gpbjnl/qzaf035)
Supplement: qzaf035_Supplementary_Data [file qzaf035_supplementary_data.zip › qzaf035_Supplementary_Data.zip!/Figure S7.pdf]

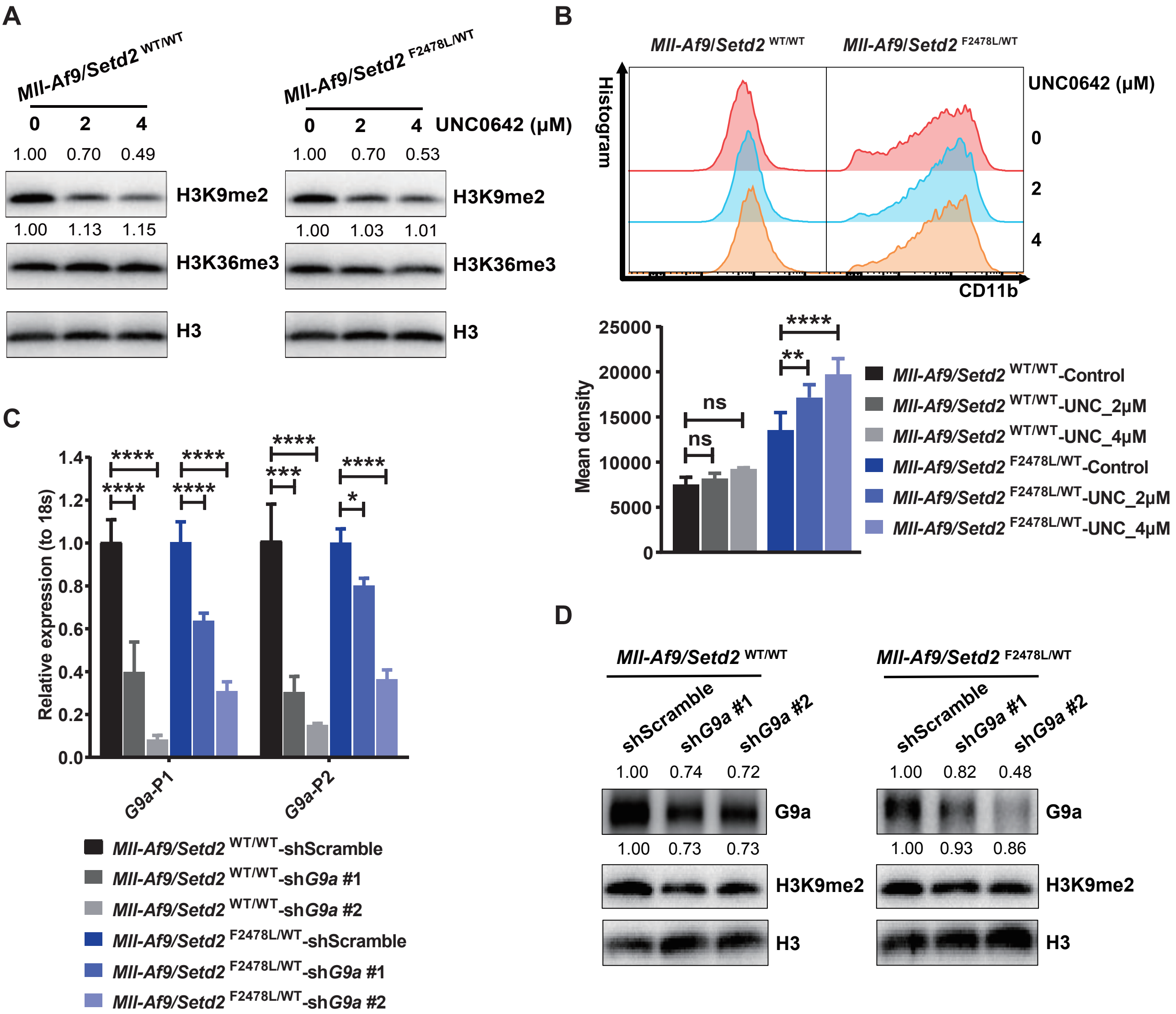
A
Mll-Af9/Setd2 WT/WT
Mll-Af9/Setd2 F2478L/WT
0 2 4
0 2 4 UNC0642 (μM)
1.00 0.70 0.49
1.00 0.70 0.53
H3K9me2
1.00 1.13 1.15
1.00 1.03 1.01
H3K36me3
H3
B
Mll-Af9/Setd2 WT/WT
Mll-Af9/Setd2 F2478L/WT
UNC0642 (μM)
0
2
4
Histogram
CD11b
Mean density
25000
20000
15000
10000
5000
0
ns
ns
****
**
Mll-Af9/Setd2 WT/WT-Control
Mll-Af9/Setd2 WT/WT-UNC_2μM
Mll-Af9/Setd2 WT/WT-UNC_4μM
Mll-Af9/Setd2 F2478L/WT-Control
Mll-Af9/Setd2 F2478L/WT-UNC_2μM
Mll-Af9/Setd2 F2478L/WT-UNC_4μM
C
Relative expression (to 18s)
1.4
1.2
1.0
0.8
0.6
0.4
0.2
0.0
****
****
****
****
****
***
****
*
G9a-P1
G9a-P2
Mll-Af9/Setd2 WT/WT-shScramble
Mll-Af9/Setd2 WT/WT-shG9a #1
Mll-Af9/Setd2 WT/WT-shG9a #2
Mll-Af9/Setd2 F2478L/WT-shScramble
Mll-Af9/Setd2 F2478L/WT-shG9a #1
Mll-Af9/Setd2 F2478L/WT-shG9a #2
D
Mll-Af9/Setd2 WT/WT
Mll-Af9/Setd2 F2478L/WT
shScramble
shG9a #1
shG9a #2
shScramble
shG9a #1
shG9a #2
1.00 0.74 0.72
1.00 0.82 0.48
G9a
1.00 0.73 0.73
1.00 0.93 0.86
H3K9me2
H3
