# Supplementary material for: Integrated Computational and Functional Screening Identifies G9a Inhibitors for SETD2-mutant Leukemia
Source: Genomics Proteomics Bioinformatics. 2025 Apr 29;23(2):qzaf035. doi: 10.1093/gpbjnl/qzaf035 (PMC12373973; doi:10.1093/gpbjnl/qzaf035)
Supplement: qzaf035_Supplementary_Data [file qzaf035_supplementary_data.zip › qzaf035_Supplementary_Data.zip!/Figure S8.pdf]

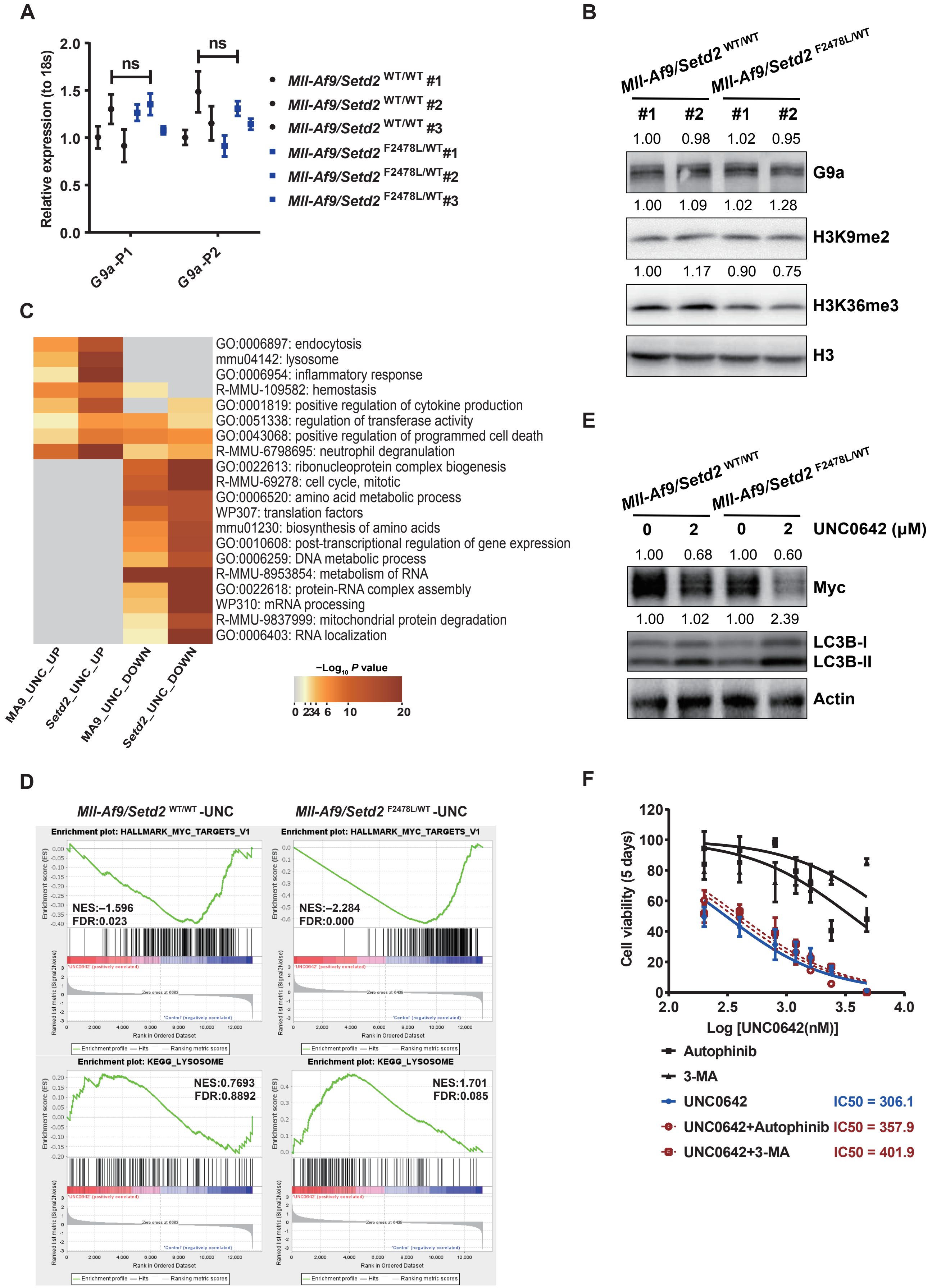
A
B
C
D
E
F
ns
ns
Relative expression (to 18s)
2.0
1.5
1.0
0.5
0.0
G9a-P1
G9a-P2
Mll-Af9/Setd2 WT/WT #1
Mll-Af9/Setd2 WT/WT #2
Mll-Af9/Setd2 WT/WT #3
Mll-Af9/Setd2 F2478L/WT #1
Mll-Af9/Setd2 F2478L/WT #2
Mll-Af9/Setd2 F2478L/WT #3
Mll-Af9/Setd2 WT/WT
Mll-Af9/Setd2 F2478L/WT
#1 #2 #1 #2
1.00 0.98 1.02 0.95
G9a
1.00 1.09 1.02 1.28
H3K9me2
1.00 1.17 0.90 0.75
H3K36me3
H3
GO:0006897: endocytosis
mmu04142: lysosome
GO:0006954: inflammatory response
R-MMU-109582: hemostasis
GO:0001819: positive regulation of cytokine production
GO:0051338: regulation of transferase activity
GO:0043068: positive regulation of programmed cell death
R-MMU-6798695: neutrophil degranulation
GO:0022613: ribonucleoprotein complex biogenesis
R-MMU-69278: cell cycle, mitotic
GO:0006520: amino acid metabolic process
WP307: translation factors
mmu01230: biosynthesis of amino acids
GO:0010608: post-transcriptional regulation of gene expression
GO:0006259: DNA metabolic process
R-MMU-8953854: metabolism of RNA
GO:0022618: protein-RNA complex assembly
WP310: mRNA processing
R-MMU-9837999: mitochondrial protein degradation
GO:0006403: RNA localization
MA9_UNC_UP
Setd2_UNC_UP
MA9_UNC_DOWN
Setd2_UNC_DOWN
−Log10 P value
0 2 3 4 6 10 20
Mll-Af9/Setd2 WT/WT -UNC
Mll-Af9/Setd2 F2478L/WT -UNC
Enrichment plot: HALLMARK_MYC_TARGETS_V1
NES:−1.596
FDR:0.023
NES:−2.284
FDR:0.000
Enrichment plot: KEGG_LYSOSOME
NES:0.7693
FDR:0.8892
NES:1.701
FDR:0.085
Enrichment score (ES)
Ranked list metric (Signal2Noise)
Rank in Ordered Dataset
Enrichment profile — Hits — Ranking metric scores
0 2 0 2 UNC0642 (μM)
1.00 0.68 1.00 0.60
Myc
1.00 1.02 1.00 2.39
LC3B-I
LC3B-II
Actin
Cell viability (5 days)
120
100
80
60
40
20
0
2.0 2.5 3.0 3.5 4.0
Log [UNC0642(nM)]
Autophinib
3-MA
UNC0642 IC50 = 306.1
UNC0642+Autophinib IC50 = 357.9
UNC0642+3-MA IC50 = 401.9
