# Supplementary material for: Integrated Computational and Functional Screening Identifies G9a Inhibitors for SETD2-mutant Leukemia
Source: Genomics Proteomics Bioinformatics. 2025 Apr 29;23(2):qzaf035. doi: 10.1093/gpbjnl/qzaf035 (PMC12373973; doi:10.1093/gpbjnl/qzaf035)
Supplement: qzaf035_Supplementary_Data [file qzaf035_supplementary_data.zip › qzaf035_Supplementary_Data.zip!/Figure S9.pdf]

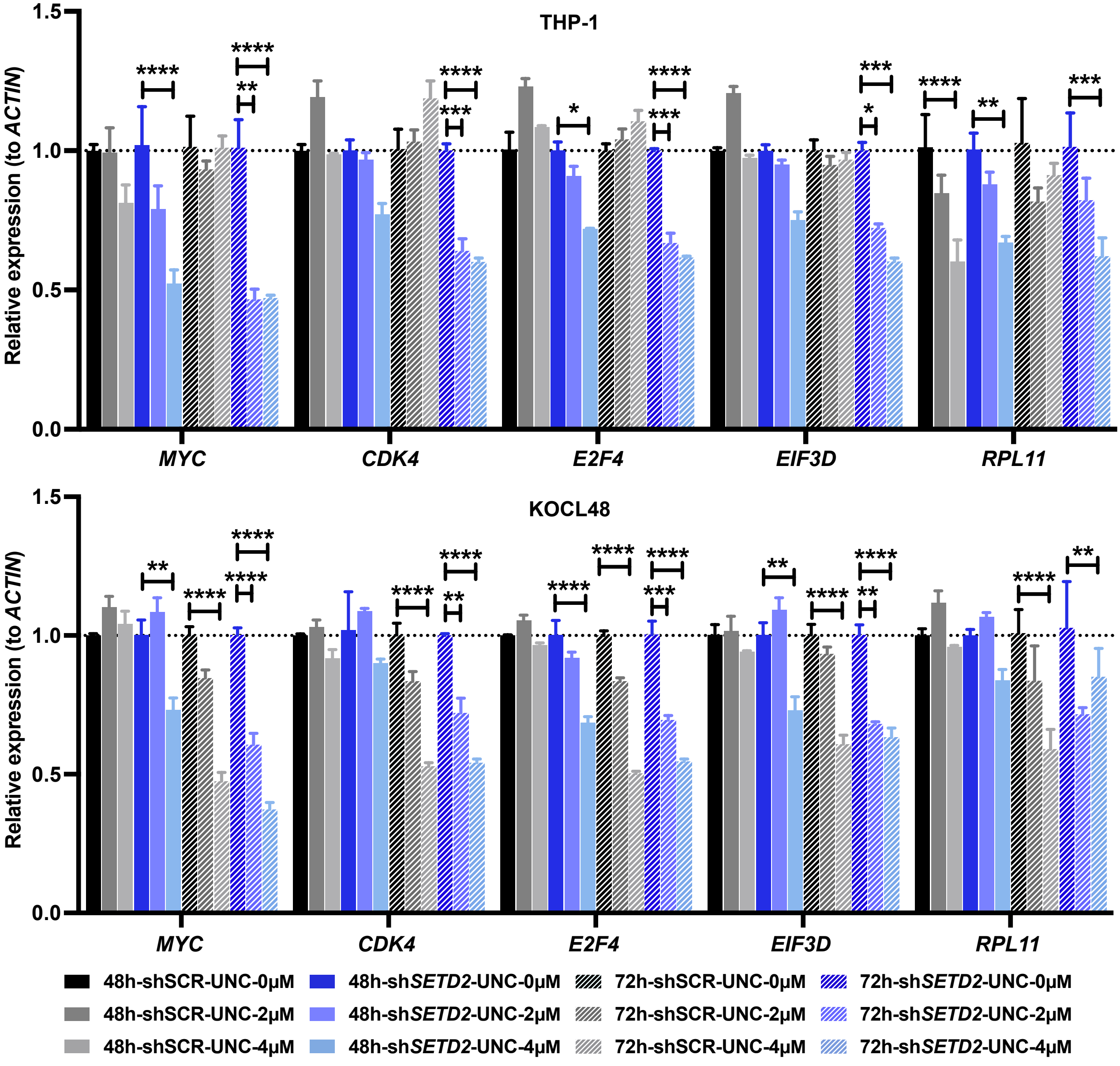

THP-1
KOCL48
Relative expression (to ACTIN)
1.5
1.0
0.5
0.0
MYC
CDK4
E2F4
EIF3D
RPL11
48h-shSCR-UNC-0μM
48h-shSCR-UNC-2μM
48h-shSCR-UNC-4μM
48h-shSETD2-UNC-0μM
48h-shSETD2-UNC-2μM
48h-shSETD2-UNC-4μM
72h-shSCR-UNC-0μM
72h-shSCR-UNC-2μM
72h-shSCR-UNC-4μM
72h-shSETD2-UNC-0μM
72h-shSETD2-UNC-2μM
72h-shSETD2-UNC-4μM
