# Supplementary material for: Integrated Computational and Functional Screening Identifies G9a Inhibitors for SETD2-mutant Leukemia
Source: Genomics Proteomics Bioinformatics. 2025 Apr 29;23(2):qzaf035. doi: 10.1093/gpbjnl/qzaf035 (PMC12373973; doi:10.1093/gpbjnl/qzaf035)
Supplement: qzaf035_Supplementary_Data [file qzaf035_supplementary_data.zip › qzaf035_Supplementary_Data.zip!/Figure S10.pdf]

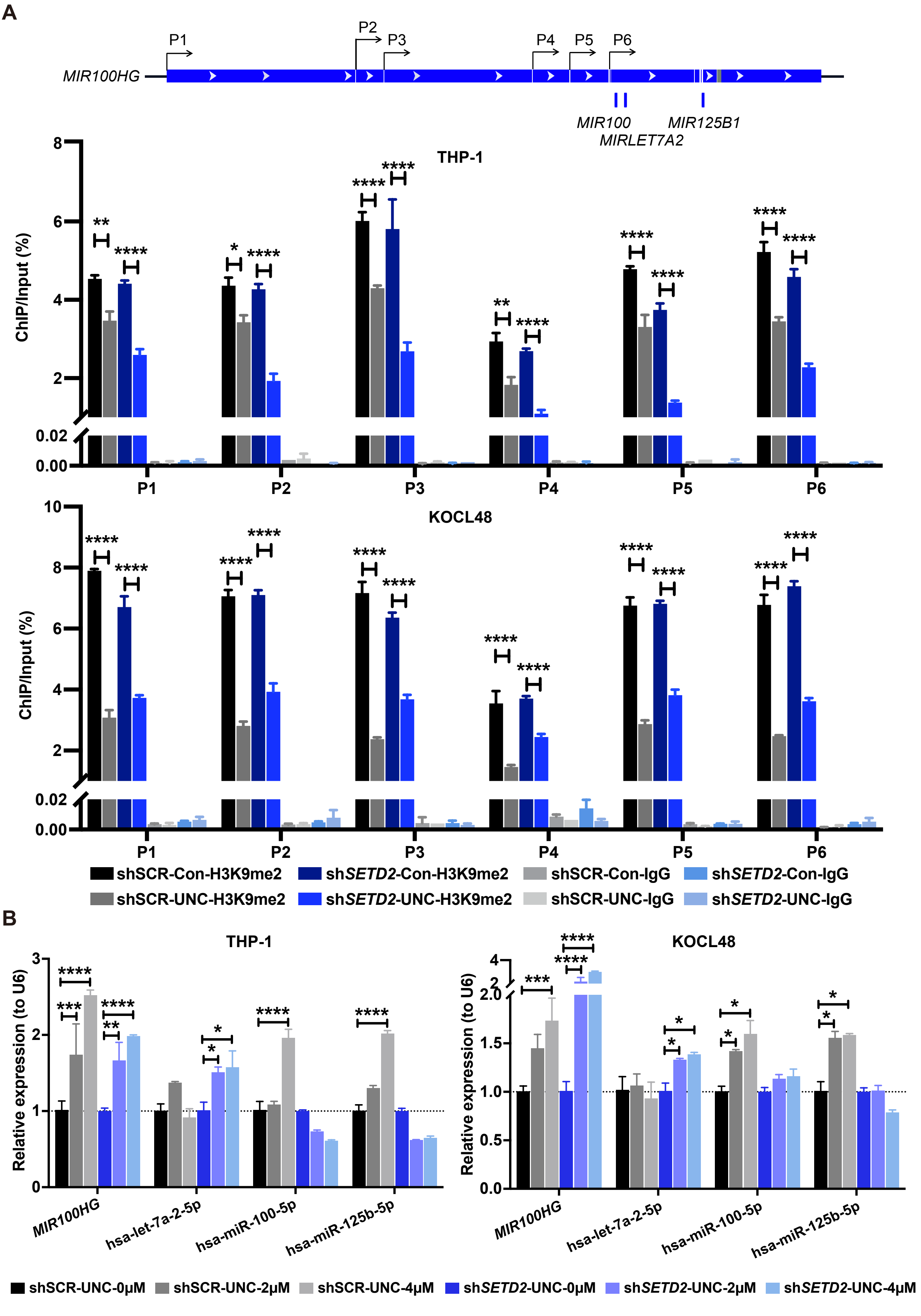
A
P1
P2
P3
P4
P5
P6
MIR100HG
MIR100
MIRLET7A2
MIR125B1
THP-1
ChIP/Input (%)
KOCL48
shSCR-Con-H3K9me2
shSETD2-Con-H3K9me2
shSCR-Con-IgG
shSETD2-Con-IgG
shSCR-UNC-H3K9me2
shSETD2-UNC-H3K9me2
shSCR-UNC-IgG
shSETD2-UNC-IgG
B
THP-1
KOCL48
Relative expression (to U6)
MIR100HG
hsa-let-7a-2-5p
hsa-miR-100-5p
hsa-miR-125b-5p
shSCR-UNC-0μM
shSCR-UNC-2μM
shSCR-UNC-4μM
shSETD2-UNC-0μM
shSETD2-UNC-2μM
shSETD2-UNC-4μM
