# Supplementary material for: Integrated Computational and Functional Screening Identifies G9a Inhibitors for SETD2-mutant Leukemia
Source: Genomics Proteomics Bioinformatics. 2025 Apr 29;23(2):qzaf035. doi: 10.1093/gpbjnl/qzaf035 (PMC12373973; doi:10.1093/gpbjnl/qzaf035)
Supplement: qzaf035_Supplementary_Data [file qzaf035_supplementary_data.zip › qzaf035_Supplementary_Data.zip!/Figure S11.pdf]

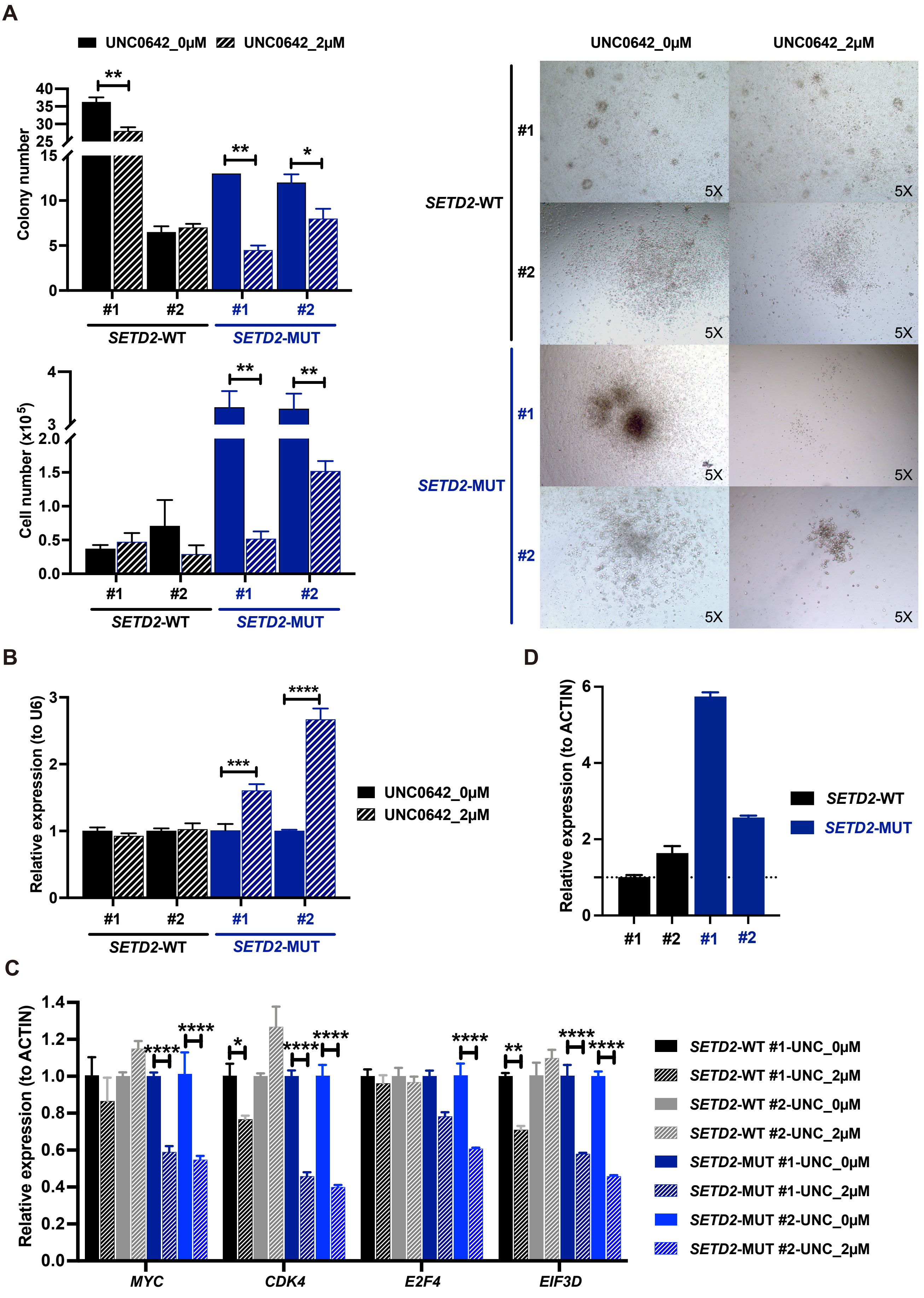
A
UNC0642_0μM
UNC0642_2μM
Colony number
40
35
30
25
15
10
5
0
**
**
*
#1
#2
#1
#2
SETD2-WT
SETD2-MUT
Cell number (x10^5)
4
3
2.0
1.5
1.0
0.5
0.0
**
**
#1
#2
#1
#2
SETD2-WT
SETD2-MUT
UNC0642_0μM
UNC0642_2μM
#1
#2
SETD2-WT
#1
#2
SETD2-MUT
5X
B
Relative expression (to U6)
3
2
1
0
***
****
#1
#2
#1
#2
SETD2-WT
SETD2-MUT
UNC0642_0μM
UNC0642_2μM
D
Relative expression (to ACTIN)
6
4
2
0
#1
#2
#1
#2
SETD2-WT
SETD2-MUT
C
Relative expression (to ACTIN)
1.4
1.2
1.0
0.8
0.6
0.4
0.2
0.0
*
**
****
MYC
CDK4
E2F4
EIF3D
SETD2-WT #1-UNC_0μM
SETD2-WT #1-UNC_2μM
SETD2-WT #2-UNC_0μM
SETD2-WT #2-UNC_2μM
SETD2-MUT #1-UNC_0μM
SETD2-MUT #1-UNC_2μM
SETD2-MUT #2-UNC_0μM
SETD2-MUT #2-UNC_2μM
