# Supplementary material for: Integrated Computational and Functional Screening Identifies G9a Inhibitors for SETD2-mutant Leukemia
Source: Genomics Proteomics Bioinformatics. 2025 Apr 29;23(2):qzaf035. doi: 10.1093/gpbjnl/qzaf035 (PMC12373973; doi:10.1093/gpbjnl/qzaf035)
Supplement: qzaf035_Supplementary_Data [file qzaf035_supplementary_data.zip › qzaf035_Supplementary_Data.zip!/supplementary material captions.docx]

**Supplementary material**

**Figure S1 *Setd2*-deficient leukemia signatures and chemoresistance characteristics revealed by RGES**

**A.** and **B.** Metascape bar graphs displaying top non-redundant biological functions enriched by upregulated (up) and downregulated (down) genes in *Mll-Af9*/*Setd2*^F2478L/WT^ cells versus *Mll-Af9* and *Mll-Af9* with *Setd2* knockdown versus scramble control. **C.** and **D.** Barcode plots showing RGES distribution of chemotherapies daunorubicin and doxorubicin calculated against *Setd2* mutant (C) and knockdown (D) leukemia signatures. A more positive score indicates a greater similarity between the drug expression profile and the disease signature.

**Figure S2 sRGES distribution of predicted candidate drug classes**

**A.** and **B.** Barcode plots showing sRGES distribution of member drugs belonging to ATM/ATR, BTK, BET and G9a inhibitors calculated against *Setd2*-mutant (A) and knockdown (B) leukemia signatures. A more negative score suggests a stronger reversal of the disease signature.

**Figure S3 Gene reversal patterns of the predicted drugs against the *Setd2*-deficient leukemia signature**

**A.** Heatmap of gene expression of *Setd2-*mutant (top) and knockdown (bottom) transcriptomic signatures, rank-ordered and color-coded (red for up, blue for down) by FC, then re-color-coded by FC rank after drug treatment as revealed by transcriptional profiles downloaded from L1000. Each column represents a drug, with daunorubicin and doxorubicin as representatives of chemotherapies, and others under each drug class corresponding to Figure S2. **B.** Enrichment network visualization for the biology functions of genes reversed by the candidate drugs against the *Setd2-*knockdown leukemia signature. Nodes are depicted as pie charts indicating their associations with each drug class. Cluster labels were added manually, and colors represent the identities of each drug class.

**Figure S4 Clustering analysis of compound library screening**

**A.** and **C.** Heatmap showing the sensitivity of *Mll-Af9*/*Setd2*^WT/WT^ and *Mll-Af9*/*Setd2*^F2478L/WT^ cells to different concentrations (0.05, 0.2, 1, 2, and 5 µM) of compounds after 5 days of treatment. Clustering analysis was performed using different distance methods: (A) Euclidean distance, (B) Maximum distance, and (C) Manhattan distance. Labels A and B represent different tree branches, and numbers indicate the main clusters under each branch. **D.** Sankey plot illustrating the flow of main clusters between ‘Euclidean distance’ and the other two methods. Clusters A2 and B1 from the Euclidean distance analysis were grouped together in Figure 2B to better represent the primary inhibition patterns.

**Figure S5 Sensitivity differences between *Setd2*-mutant and wide-type leukemic cells to the selected drugs in Figure 2C**

**A.** and **F.** Graphs showing the drug-response curves and IC50 values of (A) BET, (B) DNMT, (C) class I HDAC, (D) PARP, (E) PRMT5, (F) Topo inhibitors in *Mll-Af9* and *Mll-Af9*/*Setd2*^F2478L/WT^ leukemic cells treated with a range of concentrations for 5 days. Three representative drugs of each drug class were selected for shown. Two biological replicates of each genotype were performed, and IC50 values for each cell line are shown, with normal c-kit^+^ HSPCs as cytotoxicity controls. Mean ± SEM (*n* = 3) are shown. Topo, topoisomerase.

**Figure S6 Sensitivity differences between *Setd2*-mutant and wide-type leukemic cells to ATM/ATR-i and BTK-i and validation of *Setd2*-deficifient cells**

**A.** and **B.** Graphs showing the drug-response curves and IC50 values of (A) ATM/ATR and (B) BTK inhibitors in *Mll-Af9* and *Mll-Af9*/*Setd2*^F2478L/WT^ leukemic cells treated with a range of concentrations for 5 days. Two biological replicates were performed for each genotype, and IC50 values are shown for each cell line, with normal c-kit^+^ HSPCs as cytotoxicity controls. **C.** Representative Western blot image showing protein levels of SETD2 and H3K36me3 in human THP-1 and KOCL48 cells with sh*SETD2* and scramble control. **D.** Graphs showing dose-response curves and IC50 values of Ara-C in THP-1/KOCL48 with scrambled control or sh*SETD2*, treated with variable concentrations for 72 hours. **E.** Representative Western blot image showing protein levels of H3K36me3 in *Mll-Af9* and *Mll-Af9*/*Setd2*^F2478L/WT^ leukemic cells. Mean ± SEM (*n* = 3) are shown in A, B, and D.

**Figure S7 On-target effects of UNC0642 and G9a knockdown**

**A.** Representative Western blot images showing protein levels of H3K9me2 and H3K36me3 in *Mll-Af9* and *Mll-Af9*/*Setd2*^F2478L/WT^ cells treated with 2 µM and 4 µM UNC0642 or DMSO control for 72 hours (*n* = 3). **B.** (Top) Representative histogram showing CD11b levels in *Mll-Af9* and *Mll-Af9*/*Setd2*^F2478L/WT^ cells treated with 2 µM and 4 µM UNC0642 or DMSO control for 72 hours (*n* = 3). (Bottom) Graphical representation of the corresponding statistical results. **C.** and **D.** (C) Bar plot showing the mRNA levels of *G9a* and (D) Representative Western blot images showing protein levels of G9a and H3K9me2 in *Mll-Af9* and *Mll-Af9*/*Setd2*^F2478L/WT^ cells with scramble control and two sh*G9a* (*n* = 3). Band intensities were quantified relative to H3 and normalized to the DMSO control in B and D. P1 and P2 represented two different primer pairs in C. Mean ± SEM (*n* = 3) are shown in B and C. *, *P* < 0.05; **, *P* < 0.01; ***, *P* < 0.001; ****, *P* < 0.0001; ns, not significant; Two-way ANOVA test.

**Figure S8 G9a expression and H3K9me2 levels in *Setd2*-mutant and wild-type leukemic cells, and gene expression profiles after UNC0642 treatment**

**A.** and **B.** (A) Dot plot showing mRNA levels of *G9a* and (B) Representative Western blot image showing protein levels of G9a and H3K9me2 in *Mll-Af9* and *Mll-Af9*/*Setd2*^F2478L/WT^ cells (*n* = 3). P1 and P2 represented two different primer pairs in A. Band intensities were quantified relative to H3 and normalized to the DMSO control in B. **C.** Heatmap visualization of Metascape pathways enriched by upregulated and downregulated genes in *Mll-Af9* and *Mll-Af9*/*Setd2*^F2478L/WT^ cells after UNC0642 treatment. **D.** GSEA plots showing (left) significant (FDR ≤ 0.05) negative enrichment of MYC target gene sets and positive enrichment of lysosome gene sets in *Mll-Af9*/*Setd2*^F2478L/WT^ cells and (right) enrichment of the corresponding gene sets in *Mll-Af9* cells after UNC0642 treatment. NES, normalized enrichment score. **E.** Representative Western blot image showing protein levels of Myc and LC3B in *Mll-Af9 and Mll-Af9*/*Setd2*^F2478L/WT^ cells treated with 2 µM UNC0642 or DMSO control (*n* = 3). **F.** Graphs showing dose-response curves of UNC0642, autophagy inhibitor Autophinib and 3-MA, and their combinations in *Mll-Af9*/*Setd2*^F2478L/WT^ cells treated with variable concentrations for 5 days. Mean ± SEM (*n* = 3) are shown in A and F. ns, not significant; *t* test. FDR, false discovery rate; 3-MA, 3-methyladenine.

**Figure S9 Expression of *MYC* and *MYC* target genes in human cell lines after UNC0642 treatment**

Bar plot showing the expression levels of *MYC* and *MYC* target genes in *SETD2*-knockdown and scrambled control THP-1 (top) as well as KOCL48 (bottom) cell lines after treatment with 2 µM and 4 µM UNC0642 for 48 hours and 72 hours, relative to DMSO control. Mean ± SEM (*n* = 3) are shown. *, *P* < 0.05; **, *P* < 0.01; ***, *P* < 0.001; ****, *P* < 0.0001; Two-way ANOVA test.

**Figure S10 H3K9me2/*MIR100HG*/let-7a-2 changes in human cell lines after UNC0642 treatment**

**A.** THP and KOCL48 cells with *SETD2* knockdown or scramble control were treated with 2 µM UNC0642 or DMSO control for 72 hours. Schematic representation of the *MIR100HG* genomic locus (top) and bar plot showing the decrease in H3K9me2 levels at six potential promoter sites of *MIR100HG* (bottom). **B.** Bar plot showing the expression changes of *MIR100HG* and the mature forms of its embedded miRNAs (hsa-let-7a-5p, hsa-miR-100-5p and hsa-miR-125b-5p) after treatment with 2 µM and 4 µM UNC0642 for 24 hours, relative to DMSO control. Mean ± SEM (*n* = 3) are shown. *, *P* < 0.05; **, *P* < 0.01; ***, *P* < 0.001; ****, *P* < 0.0001; Two-way ANOVA test. miRNAs, microRNAs; ChIP, chromatin immunoprecipitation.

**Figure S11 *In vitro* UNC0642 treatment in primary patient leukemic cells**

Colony-forming assays were performed with 2 µM UNC0642 treatment and DMSO control in primary leukemic cells from *SETD2-*mutant and wild-type patients. **A.** Bar plot (left) showing colony number and total cell number counts, along with representative images (right) of the colonies. **B.** and **C.** Bar plots showing expression changes of hsa-let-7a-2-5p in (B) and *MYC* with *MYC* target genes in C after UNC0642 treatment relative to DMSO control. **D.** Bar plot showing RNA expression of *MYC* in *SETD2-*mutant and wild-type cells, and data was normalized to *SETD2* wild-type patient No. 1.

**Table S1 *Setd2*-deficient leukemia signatures**

**Table S2 sRGES and enrichment scores of drugs in the L1000 database**

**Table S3 Epigenetic compound library screening results and concentrations of tested drugs in dose-response curves**

**Table S4 DEGs in *Setd2*-mutant and wild-type cells after UNC0642 treatment**

**Table S5 Reversed DEGs by UNC0642 in *Setd2*-mutant cells against its signature, with functions enriched and upstream regulators predicted by IPA**

**Table S6 Genes with reduced H3K9me2 in *Setd2*-mutant and wild-type cells after UNC0642 treatment**

**Table S7 Upstream regulators of DEGs in *Setd2*-mutant and wild-type cells after UNC0642 treatment predicted by IPA**

**Table S8 Primers and shRNAs in this study**

**Table S9 Patient information for primary cells used in this study**
