# Supplementary material for: Integrated Computational and Functional Screening Identifies G9a Inhibitors for SETD2-mutant Leukemia
Source: Genomics Proteomics Bioinformatics. 2025 Apr 29;23(2):qzaf035. doi: 10.1093/gpbjnl/qzaf035 (PMC12373973; doi:10.1093/gpbjnl/qzaf035)
Supplement: qzaf035_Supplementary_Data [file qzaf035_supplementary_data.zip › qzaf035_Supplementary_Data.zip!/Table S9.docx]

| **Patient name** | **Age** | **Gender** | **FAB** | **Sample** | **Mutation / Fusion** |
| --- | --- | --- | --- | --- | --- |
| *SETD2* MUT Patient #1 | 54 | Female | M5 | Peripheral blood | MLL-AF9, *SETD2* |
| *SETD2* MUT Patient #2 | not known | Male | CLL | Peripheral blood | *BRCA2*, *KMT2D*, *SETD2*, *ZEB2* |
| *SETD2* WT Patient #1 | 50 | Male | M5 | Bone marrow | MLL-AF6 |
| *SETD2* WT Patient #2 | 59 | Female | Unclassified | Bone marrow | *NRAS*, *PTPN11*, *SF3B1*, *RUNX1*, *DNMT3A* |

**Table S9 Patient information for primary cells used in this study**
